# IRF4 is required for migration of CD4^+^ T cells to the intestine but not for Th2 and Th17 cell maintenance

**DOI:** 10.3389/fimmu.2023.1182502

**Published:** 2023-07-03

**Authors:** Constantin Schmidt, Aenne Harberts, Daniel Reimers, Tabea Bertram, Leonie Caroline Voß, Joanna Schmid, Niels Christian Lory, Michael Spohn, Friedrich Koch-Nolte, Samuel Huber, Friederike Raczkowski, Minka Breloer, Hans-Willi Mittrücker

**Affiliations:** ^1^ Department for Immunology, University Medical Center Hamburg-Eppendorf, Hamburg, Germany; ^2^ I. Department of Medicine, University Medical Center Hamburg-Eppendorf, Hamburg, Germany; ^3^ Clinic of Pediatric Hematology and Oncology, University Medical Center Hamburg-Eppendorf, Hamburg, Germany; ^4^ Research Institute Children’s Cancer Center Hamburg, Hamburg, Germany; ^5^ Bioinformatics Core Unit, University Medical Center Hamburg-Eppendorf, Hamburg, Germany; ^6^ Section for Molecular Biology and Immunology, Bernhard Nocht Institute for Tropical Medicine, Hamburg, Germany; ^7^ Department for Biology, University Hamburg, Hamburg, Germany

**Keywords:** Interferon Regulatory Factor 4, Th2 cells, Th17 cells, *Citrobacter rodentium*, *Strongyloides ratti*, infection

## Abstract

The transcription factor Interferon Regulatory Factor 4 (IRF4) is central in control of T cell activation and differentiation. Deficiency of IRF4 results in severe immune deficiency and affects maturation and function of most if not all T cell subsets. Here we use mouse infection models for *Citrobacter rodentium* and *Strongyloides ratti* to analyze the function of IRF4 in T helper (Th) 17 and Th2 cell responses, respectively. IRF4 deficient mice were impaired in the control of both pathogens, failed to mount Th17 and Th2 cell responses and showed impaired recruitment of T helper cells to the intestine, the infection site of both pathogens. Compromised intestinal migration was associated with reduced expression of the intestinal homing receptors α4β7 integrin, CCR9 and GPR15. Identification of IRF4 binding sites in the gene loci of these receptors suggests a direct control of their expression by IRF4. Competitive T cell transfer assays further demonstrated that loss of one functional *Irf4* allele already affected intestinal accumulation and Th2 and Th17 cell generation, indicating that lower IRF4 levels are of disadvantage for Th2 and Th17 cell differentiation as well as their migration to the intestine. Conversion of peripheral CD4^+^ T cells from an *Irf4* wildtype to an *Irf4* heterozygous or from an *Irf4* heterozygous to a homozygous mutant genotype after *C. rodentium* or *S. ratti* infection did not reduce their capacity to produce Th17 or Th2 cytokines and only partially affected their persistence in the intestine, revealing that IRF4 is not essential for maintenance of the Th2 and Th17 phenotype and for survival of these T helper cells in the intestine. In conclusion, we demonstrate that the expression levels of IRF4 determine Th2 and Th17 cell differentiation and their intestinal accumulation but that IRF4 expression is not crucial for Th2 and Th17 cell survival.

## 1 Introduction

The transcription factor Interferon Regulatory Factor 4 (IRF4) is expressed in cells of the immune system, including B and T cells as well as subsets of macrophages, dendritic cells (DC) and innate lymphoid cells such as innate lymphoid type-2 cells (ILC2) (1–3). Naive T cells express only low levels of IRF4, however, upon T cell receptor stimulation IRF4 is induced and rapidly expressed at high levels. Several DNA sequence motifs recognized by IRF4 have been identified. IRF4 can bind to interferon stimulated response elements (ISRE). In complex with the transcription factors PU.1 and SpiB, it interacts with Ets-IRF composite elements (EICE) and in complexes with BATF and heterodimers of Jun family members with activator protein 1 (AP-1)-IRF composite elements (AICE) (4–7). In Th17 and CD8^+^ T cells, large numbers of IRF4 regulated genes have been identified. IRF4 targets include genes involved in T cell differentiation and effector functions but also genes involved in more fundamental cellular processes such as metabolism and proliferation. IRF4 binding sites are frequently located within regulatory DNA regions distant from promotors. IRF4 is therefore also considered as a pioneering factor that promotes and sustains chromatin remodeling and thereby enhances accessibility of genes for other transcription factors (8–10).

Due to its essential role in regulation of fundamental cellular processes and its cooperation with lineage-specific transcription factors, IRF4 is essential for effective activation of peripheral CD4^+^ and CD8^+^ T cells and their differentiation into various effector T cell subsets. Accordingly, mice lacking IRF4 in T cells are impaired in mounting peripheral responses of basically all T cell lineages including, CD4^+^ T helper (Th) 1, Th2, Th17, T follicular helper (Tfh), and T regulatory (Treg) cells as well as CD8^+^ cytotoxic T cells (11–18). Due to this central role in T cells and similar essential functions in B cells, homozygous mutation of IRF4 causes severe immune deficiency in mice and human (19–21). Expression of IRF4 directly correlates with the strength of the TCR signal. IRF4 binding motifs differ in their affinity for IRF4 and thus the selection of targeted genes depends on the concentration of IRF4. Therefore, IRF4 translates the strength of the TCR signal into distinct fates of T cell differentiation (10, 14). In line with this mechanism, mutation of one *Irf4* alleles already results in diminished CD8^+^ and CD4^+^ Th1 cell responses to pathogens (22–25).

Following T cell activation, IRF4 is only transiently upregulated, and expression decreases to low level in memory T cells. In chronic infection models, CD8^+^ T cells heterozygous for *Irf4* mutation are protected from exhaustion and in mouse tumor models, disruption of the AICE binding complex prevents development of exhaustion in CAR T cells. Thus, at later stages of the T cell response reduction of IRF4 activity appears to be required to sustain functionality of CD8^+^ T cells (9, 24, 26). The role of IRF4 in memory T cells is rather unclear mainly due to the inefficient memory cell development of IRF4 deficient T cells. Using a mouse model that allows deletion of *Irf4* allele at defined time points, we could recently demonstrate that deletion of *Irf4* after T cell activation does not compromise survival of CD8^+^ memory T cells and their production of cytokines in response to stimulation. However, upon reencounter of the pathogen, these CD8^+^ T cells show impaired expansion and effector functions. In contrast to CD8^+^ effector memory T cells, CD8^+^ tissue-resident T (Trm) cells express higher levels of IRF4 protein and induced deletion of *Irf4* alleles in peripheral T cells results in a reduction of these CD8^+^ Trm cells (18, 25).

Here, we use mouse models for intestinal nematode infection (*Strongyloides ratti*) and intestinal bacterial infection (*Citrobacter rodentium*) to analyze the Th2 and Th17 cell responses of *Irf4*-deficient CD4^+^ T cells. Mice with ubiquitous deficiency in *Irf4* were impaired in the control of both pathogens and completely failed to mount Th2 and Th17 cell responses. We also observed diminished accumulation of CD4^+^ T cells in the intestinal tract of *Irf4* deficient mice, which was at least in part due to compromised expression of intestinal homing receptors α4β7, CCR9 and GPR15. After competitive T cell transfer, CD4^+^ T cells heterozygous for *Irf4* mutation were already impaired in generating Th2 and Th17 cells responses to infection and in their accumulation in the intestinal tract of recipient mice. Induced deletion of the remaining *Irf4* allele after infection further reduced frequencies of intestinal CD4^+^ T cells in the colon but not in the small intestine. Thus, IRF4 appears to be essential for migration of CD4^+^ T cell into the intestine but less important for their persistence in this tissue. Induced *Irf4* deletion after infection did not further reduce the frequencies of IL-4^+^, IL-13^+^ or IL-17A^+^ T cells indicating that IRF4 is not essential for production of these cytokines in Th2 and Th17 memory cells.

## 2 Materials and methods

### 2.1 Mice

*Irf4* ^-/-^ mice (B6.129P2-*Irf4* ^tm1Mak^/J) (21), *Irf4* ^fl/fl^ mice (20), *Rag1* ^-/-^ mice (B6.129S7-*Rag1^tm1Mom^*/J) (27), *Rosa-CreER* ^T2^ mice (28), CD45.1 congenic mice (B6.SJL-*Ptprc^a^ Pepc^b^*/BoyJ), and CD90.1 congenic mice (B6.PL-*Thy1^a^*/CyJ) were maintained on a C57BL/6 genetic background. All other mice used in experiments were derived by intercrosses of these strains. Genotypes of mice were determined by PCR as described previously (20, 21) and by flow cytometry of blood samples. All mice were bred and housed at the animal facility of the University Medical Center Hamburg-Eppendorf under specific pathogen-free conditions with standard food and water *ad libitum*. During infection experiments, mice were controlled daily and mice with signs of severe disease were eliminated to minimize suffering. For all experiments, age and sex matched groups of mice were used. All animal experiments were conducted in agreement with the German animal protection law and experimental protocols were approved by the local committee for animal experiments of the City of Hamburg (registration numbers: N017/2017, N055/2019 N068/2021, N150/2021).

### 2.2 T cell transfer and tamoxifen treatment of mice

Cells for transfer experiments were isolated from donor mice with an age of 4-10 weeks. Recipient *Rag1* ^-/-^ mice on a CD45.1 congenic background were at least 4 weeks of age when reconstituted. For transfer experiments, T cells from spleens of *Irf4* ^+/+^, *Irf4* ^+/-^, *Irf4* ^-/-^, *Irf4* ^+/fl^×*CreER* ^T2^ (CD90.1^+^ CD90.2^+^) and *Irf4* ^-/fl^×*CreER^T2^* mice (CD90.1^−^ CD90.2^+^) were purified by negative selection using the EasySep™ Mouse T Cell Isolation Kit or EasySep™ Mouse CD4^+^ T cell Isolation Kit (STEMCELL™ Technologies, Vancouver, Canada) according to the manufacturer’s protocol. Purification and subsequent mixing of cells were controlled by fluorescence-activated cell sorting (FACS) analysis. Purification consistently reached >95%. T cells from *Irf4* ^+/fl^×*CreER* ^T2^ (CD90.1^+^ CD90.2^+^) and *Irf4* ^-/fl^×*CreER* ^T2^ mice (CD90.1^−^ CD90.2^+^) were mixed in a 1:1 ratio and a total of 8×10^6^ T cells per mouse in 200 μl of sterile phosphate-buffered saline (PBS) were transferred into CD45.1^+^ *Rag1* ^−/−^ mice by tail vein injection. Transferred *Irf4* ^+/fl^×*CreER* ^T2^ and *Irf4* ^-/fl^×*CreER* ^T2^ T cells could be distinguished by CD90.1 staining. For transfer of either *Irf4^+/+^*, *Irf4^+/-^* or *Irf4* ^-/-^ T cells, a total of 3-5×10^6^ T cells per mouse in 200 μl of PBS were transferred into CD45.1^+^ *Rag1* ^−/−^ mice by tail vein injection. For activation of the Cre recombinase in mice, 2 mg of tamoxifen (Sigma Aldrich, St. Louis, MO) per day dissolved in corn oil (Sigma Aldrich) was applied intraperitoneally on 5 consecutive days. Analyses were performed at least 7 days after the last tamoxifen injection.

### 2.3 Infection of mice

The *Strongyloides ratti* cycle was maintained by serial passage in Wistar rats as described (29). For *S. ratti* infection, 2000 infectious third-stage larvae (L3) were injected subcutaneously (s.c.) in 30µl PBS into the hind footpad. Quantification of parasite burden in the intestines of infected mice was performed as described (30). For *Citrobacter rodentium* infection, mice were infected via oral gavage with 1×10^9^ colony forming units (CFU) in 200 μl sterile PBS. *C. rodentium* bacteria were grown overnight at 37°C at 200 rpm in LB medium containing nalidixic acid (50 μg/ml). The number of bacteria was determined by optical density at 600 nm (OD_600_), and the suspension was diluted with PBS to the final concentration. Bacterial inocula were controlled by plating serial dilutions on tryptic soy broth agar or LB agar plates (with the antibiotic nalidixic acid for further selection) and colonies were counted after further 2-3 d of incubation at room temperature (RT). For quantification of *C. rodentium* titers, organs were homogenized in PBS, serial dilutions of suspensions were plated on LB agar and CFU were counted after incubation for 2-3 days at RT.

### 2.4 T cell isolation and analysis

Infected mice were sacrificed, and organs harvested at indicated time points. Anti-ART2A nanobodies (clone: S+16, 50 μg in 100 μl PBS) were injected before sacrificing the mice to prevent NAD-induced cell death (31). To stain intravascular cells, 2.5 μg of PerCP-conjugated anti-CD45 monoclonal antibody (clone 30F-11, BioLegend, San Diego, CA) were injected (32). Both antibodies were administered together intravenously 3 minutes before sacrificing the mice.

For the analysis of spleen cells, spleens were forced through a 70 μm cell strainer. Erythrocytes were lysed using lysis buffer (155 mM NH_4_Cl, 10 mM KHCO_3_, 100 μM EDTA, pH 7.2) for 3 minutes. Mesenteric lymph nodes (mLN) were squashed through a 30 μm cell strainer. For isolation of intestinal cells, coli including caeci or small intestines, respectively, were harvested. Lymph nodes, Peyer’s patches and excessive adventitial fat were removed, followed by longitudinally cutting and washing of the intestinal tissues with PBS. To isolate intraepithelial lymphocytes (IEL), the organ pieces were shaken in Gibco™ Hanks’ Balanced Salt Solution (HBSS, Thermo Fisher Scientific, Waltham, MA) (without Ca^2+^ and Mg^2+^) containing 1 mM dithioerythritol for 20 min at 37°C. The supernatants containing IELs were collected. The isolation of lamina propria lymphocytes (LPL) required further dissociation, therefore the intestinal tissues were minced, forced through a 100 μm cell strainer and shaken in HBSS (without Ca^2+^ and Mg^2+^) containing collagenase (1 mg/ml, Roche, Mannheim, Germany) and DNase I (10 U/ml, Sigma-Aldrich, St. Louis, MO) for 45 min at 37°C. The supernatants containing the LPLs were collected and pooled with the supernatants containing the IELs. Cells were washed, and lymphocytes were enriched via density gradient centrifugation with two phases (40% and 70% Percoll, GE Healthcare, Chicago, IL).

For detection of intracellular cytokines, cells were incubated in RPMI 1640 medium or Iscove’s modified Dulbecco’s medium (Gibco™ RPMI 1640 or IMDM, Thermo Fisher Scientific) supplemented with 5% fetal calf serum, L-glutamine, pyruvate, gentamicin and 2-mercaptoethanol. Cells were polyclonally stimulated with phorbol 12-myristate 13-acetate (PMA, 50 ng/ml, Sigma Aldrich) and ionomycin (1 μM, Sigma Aldrich) for 4 hours at 37°C. To prevent cytokine secretion, Brefeldin A (BFA, 10 μg/ml, Sigma Aldrich) was added for the last 3.5 h of culture. Afterwards cytokine expression was determined by intracellular staining and flow cytometry.

### 2.5 ELISA

For analysis of cellular responses, *S. ratti* infected mice were sacrificed at day 6 post infection (p.i.). mLN and spleen cells were obtained by mashing the organs through cell sieves into PBS, followed by erythrocyte lysis. 1×10^6^ cells were cultured in triplicates in 96-well round-bottom plates in RPMI 1640 medium supplemented with 5% FCS, HEPES (20 mM), L-glutamine (2 mM), 2-mercaptoethanol (50 mM) and gentamicin (50 μg/mL) at 37°C and 5% CO_2_, and stimulated for 72 h with medium alone, anti-mouse CD3ϵ mAb (clone 145-2C11, 1 μg/mL, BioLegend), or *S. ratti* L3 lysate (20 μg/mL). Cytokines in the culture supernatants were quantified by ELISA, as described (30), using ELISA Kits from BioLegend (San Diego, CA). Detection limits with the standard dilutions used in this study were < 15 pg/ml for IL-4, IL-5 and < 30 pg/ml for IL-10, IL-13 and IFN-γ.

### 2.6 Antibody responses to *S. ratti* infection

Blood was collected from infected mice at the indicated time points and allowed to coagulate for 1h at RT. Serum was collected after centrifugation (10.000×g) for 10 min at RT. *S. ratti*-specific serum Ig titers were quantified by ELISA, as described (30).

### 2.7 Antibodies and flow cytometry

Single cell suspensions were incubated with PBS with 1:100 rat serum and 10 μg/mL 2.4G2 (anti-FcγRII/III, BioXCell, West Lebanon, NH) to minimize unspecific antibody binding. Viability was determined using Pacific Orange succinimidyl ester staining. Extracellular staining was performed with fluorochrome-conjugated mAbs (**Supplementary Table 1**). For intracellular staining of cells expressing eGFP, cells were fixed with 3.7% formaldehyde in PBS 1% fetal calf serum (FCS) for up to 30 min at 4°C. Cells were washed with PBS 1% FCS and permeabilized with PBS 1% FCS containing 0.1% Igepal CA-630 (Sigma Aldrich) for 5 min at RT. Cells were then washed with PBS 1% FCS and stained with mAbs in PBS 1% FCS for 20 min at RT. In cells not expressing eGFP, intracellular staining for GATA3, IRF4, RORγt, IL-4, IL-10, IL-13, IL-17A, IFN-γ and TNF-α was performed using the FoxP3 staining buffer set (eBiosciences, San Diego, CA) according to the manufacturer’s protocols. Cells were acquired with FACSCanto™ II or FACSCelesta™ flow cytometers and BD FACSDiva™ software (BD Biosciences, Heidelberg, Germany) and analyzed with FlowJo 10.8.1 software (FlowJo LLC, Ashland, OR).

### 2.8 Transcription factor binding peaks analysis

Raw *Batf* and *Irf4* ChIP-Seq reads from GSE40483 (4) were downloaded from GEO with *fastq-dump* from the sra-toolkit v2.11.0. Adapter sequences and low-quality bases at the 3’ends were removed with fastp v0.23.2 (33). Trimmed reads were then aligned to the mm10 reference genome with bowtie2 v2.3.5.1 (34) and afterwards filtered by samtools v1.10 (35) *view* for alignments with mapping quality > 2 to account for multimapping reads. Duplicate alignments were removed by samtools *rmdup* before Macs2 v2.2.7.1 (36) was employed for peak calling. Bowtie v 1.3.1 (37) was then used to map all possible versions of the ISRE GAAANNGAAA motifs, AP-1 TGANTCA motifs and IRF GAAA motif to the mm10 mouse reference genome, no mismatches were allowed and all alignments were reported. Resulting alignments were transformed into bed format with bedtools v2.30.0 (38) *bamtobed.* Only *Batf* peaks overlapping an ISRE or AP-1 motif were kept, *Irf4* peaks overlapping the IRF motif respective. Therefore, bedtools *intersect* was used with -F set to 1.0 to obtain peaks obtaining the full motif. All data was then visualized by IntegrativeGenomicsViewer (IGV) v2.16.0 (39).

### 2.9 Statistical analyses

Statistical analyses were performed using GraphPad Prism (GraphPad Software Inc., La Jolla, CA). Results were analyzed with the tests indicated in the figure legends. In the case of three or more groups, one-way analysis of variance (ANOVA) with Tukey’s multiple comparisons test as indicated was used. In T cell transfer experiments with subsequent deletion of *Irf4* alleles, GFP^+^ cells and GFP^−^ cells with deleted and non-modified alleles were detected, respectively. In these experiments, GFP^+^ and GFP^−^ CD4^+^ T cells in individual mice were matched and results of groups of mice were analyzed with paired t test. A p-value of < 0.05 was considered significant and is indicated in the graphs (*p < 0.05; **p < 0.01; ***p < 0.001; ****p < 0.0001). Values without any indication were not significant.

## 3 Results

### 3.1 IRF4-deficient mice show impaired Th17 cell response to *Citrobacter rodentium* infection

**Figure 1:**
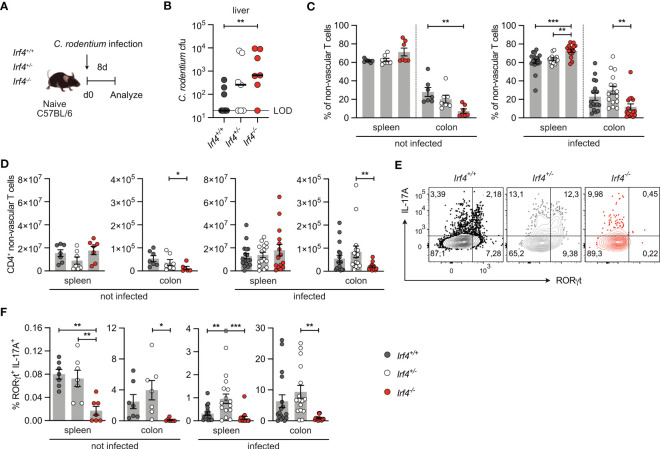
IRF4-deficient mice show impaired Th17 cell response to intestinal *Citrobacter rodentium* infection. **(A)** Experimental set up: *Irf4* ^+/+^ *, Irf4* ^+/-^ and *Irf4* ^-/-^ mice were infected with *C rodentium*. After 8 days, colon and spleen cells were isolated, stimulated with PMA and ionomycin for 4 h, and the production of different cytokines in βTCR^+^ CD4^+^ T cells was determined by intracellular cytokine staining and flow cytometry. **(B)** *C rodentium* titers in the liver (LOD = limit of detection). Individual values and median are shown, data was analyzed with Mann-Whitney test. **(C)** Percentages and **(D)** numbers of CD4^+^ T cells in spleen and colon of naive and infected mice. **(E)** Representative dot plots of IL-17A and RORγt-producing CD4^+^ T cells from colon of infected mice after stimulation with PMA and ionomycin. **(F)** Expression of RORγt and IL-17A in CD4^+^ T cells from spleen and colon of naive and infected mice after stimulation with PMA and ionomycin. **(C, F)** Pooled results of 2 independent experiments in naive groups and 3 independent experiments in infected groups. Mean ± SEM, one-way ANOVA with Tukey’s multiple comparisons test. **(D)** Pooled results of 3 independent experiments in the infected group and two experiments in the naive group. Mean ± SEM, results were analyzed with Kruskal-Wallis test and Dunn’s multiple comparisons test. (*p < 0.05; **p < 0.01; ***p < 0.001).

IRF4 has a central role in the activation and differentiation of T cells including differentiation of Th17 cells (13). Here, we use infection of mice with the enteric pathogen *C. rodentium* for induction of an intestinal Th17 cell response in mice (40, 41). *Irf4* ^+/+^, *Irf4* ^+/-^ and *Irf4* ^-/-^ mice were orally infected with *C. rodentium* (**Figure 1A**). Eight days post-infection, *Irf4* ^-/-^ mice had higher citrobacter titers in the liver, indicating that IRF4 was required for restriction of bacterial dissemination (**Figure 1B**). When compared to *Irf4* ^+/+^ and *Irf4* ^+/-^ mice, *Irf4* ^-/-^ mice had reduced percentages and numbers of CD4^+^ T cells in their colon both under homeostatic conditions and upon *C. rodentium* infection (**Figures 1C, D**, Gating strategy in **Supplementary Figure 1A**). In naive *Irf4* ^+/+^ mice, we observed after polyclonal stimulation only marginal frequencies of RORγt^+^ IL-17A^+^ Th17 cells in spleens and low frequencies in the colon (**Figures 1E, F**; **Supplementary Figures 1B, C**). Th17 cell populations strongly increased in both spleen and colon following infection of *Irf4* ^+/+^ and *Irf4* ^+/-^ mice. However, Th17 cells were hardly detectable in spleen and colon of naive and infected *Irf4* ^-/-^ mice. In contrast to Th17 cells, IFN-γ^+^ and TNF-α^+^ CD4^+^ Th1 cells were present in roughly similar frequencies in spleen and colon of *Irf4^+/+^* and *Irf4^-/-^* mice under steady state conditions as well as following infection (**Supplementary Figures 1B-F**). It has been demonstrated that the strength of IRF4 expression controls T cell activation and thus loss of one *Irf4* allele can already alter T cell differentiation (10, 22). However, following *C. rodentium* infection the Th17 cell response of *Irf4* ^+/-^ mice was similar or even stronger than that of *Irf4* ^+/+^ mice. Under homeostatic conditions, CD4^+^ T cells of *Irf4^+/+^*, *Irf4^+/-^* and *Irf4^-/-^* mice expressed similar or only slightly reduced levels of the anti-apoptotic proteins Bcl-2l1 (Bcl-X) and Bcl-2, respectively, and similar levels of the transcription factor TCF-7 associated with long term T cell survival (**Supplementary Figure 1G**), indicating that absence of one or both *Irf4* alleles did not affect T cell survival. In conclusion, these results demonstrate that IRF4 is required for intestinal Th17 cell responses to oral bacterial infection.

### 3.2 IRF4 is not required for the maintenance of Th17 cells

**Figure 2:**
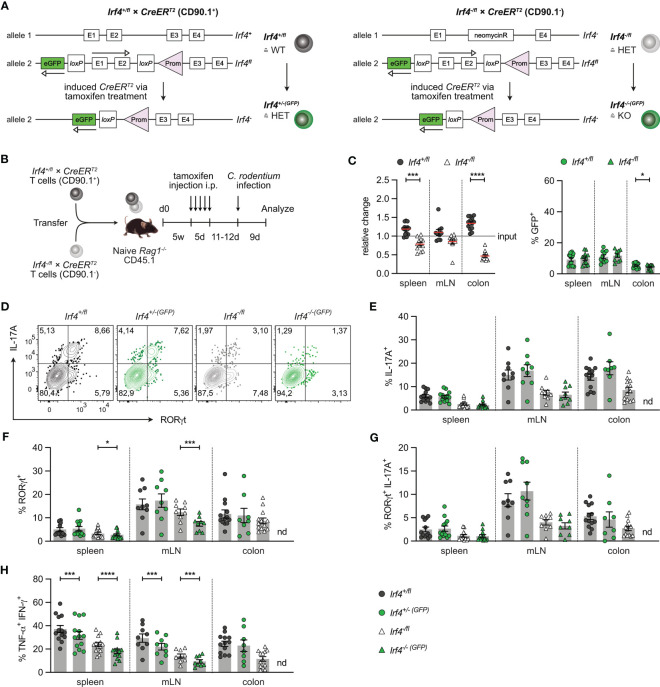
IRF4 dose dependent impairment of Th17 cell differentiation. **(A)** Scheme of genetic modifications of the *Irf4* locus induced by tamoxifen activation of Cre in *Irf4* ^+/fl^×*CreER* ^T2^ (left) and *Irf4* ^-/fl^×*CreER* ^T2^ cells (right). **(B)** Experimental set up: naive *Rag1* ^−/−^ mice were reconstituted with 4×10^5^ T cells from each naive *Irf4* ^+/fl^×*CreER* ^T2^ (CD90.1^+^) and *Irf4* ^-/f^ *^l^* ×*CreER* ^T2^ (CD90.1^-^) mice. After 4-5 weeks, recipients were treated with tamoxifen on 5 consecutive days. 11-12 days post tamoxifen treatment, mice were infected orally with *C. rodentium* and analyzed 9 days post infection. **(C)** Relative change of the *Irf4* ^+/fl^×*CreER* ^T2^ and *Irf4^-/fl^×CreER* ^T2^ CD4^+^ T cell population determined by % cells of CD4^+^ T cells at time point of analysis divided by the % cells of transferred CD4^+^ T cells (left), and % GFP^+^ cells of *Irf4* ^+/fl^×*CreER* ^T2^ and *Irf4* ^-/fl^×*CreER* ^T2^ CD4^+^ T cells in spleen, mesenteric lymph nodes (mLN) and colon (right). **(D-H)** T cells isolated from spleen, mLN and colon of infected mice were stimulated for 4h with PMA and ionomycin. **(D)** Representative FACS plots for IL-17A and RORγt of CD4^+^ T cells from mLN. **(E)** Percentages of IL-17A^+^, **(F)** RORγt^+^, **(G)** IL-17A^+^RORγt^+^ and **(H)** TNF-α^+^IFN-γ^+^ CD4^+^ T cells. **(C, E-H)** Pooled results of 2 independent experiments. For all experiments: Mean ± SEM, results were analyzed with paired t test. (*p < 0.05; ***p < 0.001; ****p < 0.0001).

IRF4 is expressed in diverse hematopoietic cells including DCs which might affect CD4^+^ T cell differentiation. To define the T cell intrinsic role of IRF4, we used a T cell transfer assay that allows knock-out of the *Irf4* gene in T cells at defined time points (25). *Rag1* ^-/-^ mice were reconstituted with T cells from *Irf4* ^+/fl^×*CreER* ^T2^ or *Irf4* ^-/fl^×*CreER* ^T2^ mice. In cells from *Irf4* ^+/fl^×*CreER* ^T2^ mice, tamoxifen treatment causes the switch from two functional alleles (*Irf4* ^+/fl^ *)* to one functional allele (*Irf4* ^+/-(GFP)^) and in cells from *Irf4* ^-/fl^×*CreER* ^T2^ mice, the switch from one functional allele (*Irf4* ^-/fl^) to a homozygous knockout genotype (*Irf4* ^-/-(GFP)^). Since Cre-mediated deletion of the *Irf4* ^fl^ allele activates GFP expression, cells with productive recombination become GFP-positive (**Figure 2A**) (20). With this approach, we usually observe GFP expression in 5-15% of CD4^+^ T cells of both donor cell populations. GFP expression correlated with a reduction of IRF4 protein expression in both cell populations, indicating the loss of one functional *Irf4* allele in these cells (**Supplementary Figure 2A**).

To test whether continuous T cell intrinsic IRF4 expression is required for Th17 cell differentiation, T cells from *Irf4* ^+/fl^×*CreER* ^T2^ and *Irf4* ^-/fl^×*CreER* ^T2^ mice were mixed in a 1:1 ratio and transferred into *Rag1* ^-/-^ mice. After 5 weeks, mice were treated with tamoxifen, and 11-12 days after the end of tamoxifen treatment, mice were orally infected with *C. rodentium*. T cells from spleen, mesenteric lymph node (mLN) and colon were characterized 9 days post-infection (**Figure 2B**). Using this protocol, we observed a disadvantage of *Irf4* ^-/fl^×*CreER* ^T2^ CD4^+^ T cells to accumulate in spleen and mLN, and a profound defect to populate the colon (**Figure 2C**; **Supplementary Figures 2B, C**). In spleen and mLN, similar frequencies of cells in both donor cell populations had converted to GFP^+^ cells. When compared to *Irf4* ^+/fl^×*CreER* ^T2^ CD4^+^ T cells from spleen and mLN, *Irf4* ^+/fl^×*CreER* ^T2^ cells in the colon had lower frequencies of GFP^+^ cells and frequencies of GFP^+^ cells were further reduced in colonic *Irf4* ^-/fl^×*CreER* ^T2^ CD4^+^ T cells. Overall, these results indicate that *Irf4* heterozygous and particularly *Irf4* homozygous mutant CD4^+^ T cells were deficient in accumulating in the colon.

T cells from spleen, mLN and colon were stimulated with PMA and ionomycin and analyzed for IL-17A and RORγt expression (**Figures 2D-G**). In all tissues, *Irf4* heterozygous *Irf4* ^-/fl^×*CreER* ^T2^ CD4^+^ T cells showed reduced percentages of IL-17A^+^ RORγt^+^ Th17 cells when compared to *Irf4* ^+/fl^×*CreER* ^T2^ cells with two functional *Irf4* alleles. Thus, under this experimental condition, deficiency of one *Irf4* allele already impaired Th17 cell development. When compared to their GFP^-^ counterparts, GFP^+^ CD4^+^ T cells of both *Irf4* ^+/fl^×*CreER* ^T2^ and *Irf4* ^-/fl^×*CreER* ^T2^ populations in spleen and mLN contained similar low percentages of IL-17A^+^ and of IL-17A^+^ RORγt^+^ cells. However, we observed a reduction in *Irf4* ^-/-(GFP)^×*CreER* ^T2^ T cells when analysis was restricted to RORγt (**Figure 2F**). Due to the very low number of *Irf4* ^-/-(GFP)^×*CreER* ^T2^ T cells recovered from the colon (<20 cells per sample), we were not able to reliably determine RORγt and cytokine expression in these cells. To determine the development of Th1 cells, we measured the expression of IFN-γ and TNF-α in CD4^+^ T cells (**Figure 2H**; **Supplementary Figure 2D**). Frequencies of Th1 cells were lower in *Irf4* ^-/fl^×*CreER* ^T2^ CD4^+^ T cells and Cre-mediated mutation of one *Irf4* allele further reduced the frequencies of Th1 cells in both GFP^+^ CD4^+^ T cells of both *Irf4* ^+/fl^×*CreER* ^T2^ and *Irf4* ^-/fl^×*CreER* ^T2^ populations in spleen and mLN. Overall, these results suggest that in an *Irf4* wildtype environment, heterozygous CD4^+^ T cells were impaired in the development of Th17 cells. Loss of an *Irf4* allele in peripheral T cells did not affect the Th17 cell response of *Irf4* ^+/-(GFP)^×*CreER* ^T2^ cells, but diminished the differentiation of *Irf4* ^-/-(GFP)^×*CreER* ^T2^ T cells to RORγt^+^ Th17 cells.

**Figure 3:**
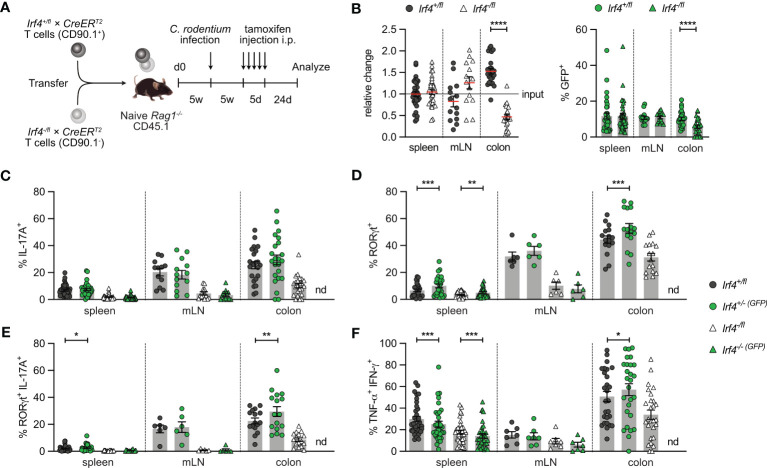
Loss of IRF4 does not prevent Th17 cell maintenance. **(A)** Experimental set up: naive *Rag1* ^−/−^ mice were reconstituted with 4×10^5^ T cells from each naive *Irf4* ^+/fl^×*CreER* ^T2^ (CD90.1^+^) and *Irf4* ^-/fl^×*CreER* ^T2^ (CD90.1^-^) mice. After reconstitution for 4-5 weeks, recipient mice were infected orally with *C. rodentium.* After further 5 weeks, mice were treated with tamoxifen (TA) for 5 days. Mice were analyzed 24 days post TA treatment. **(B)** Relative change of the *Irf4* ^+/fl^×*CreER* ^T2^ and *Irf4^-/fl^×CreER* ^T2^ CD4^+^ T cell populations determined by % cells of CD4^+^ T cells at time point of analysis divided by the % cells of transferred CD4^+^ T cells (left). % GFP^+^ cells of *Irf4* ^+/fl^×*CreER* ^T2^ and *Irf4* ^-/fl^×*CreER* ^T2^ CD4^+^ T cells in spleen, mesenteric lymph node (mLN) and colon (right). **(C-F)** Cells of infected mice from spleen, mLN and colon were stimulated with PMA and ionomycin for 4 h, and percentages of IL-17A^+^, RORγt^+^, IL-17A^+^ RORγt^+^ and TNF-α^+^ IFN-γ^+^ CD4^+^ T cells were determined. Pooled results of four independent experiments for spleen and MLN, and two independent experiments for colon in infected groups. For all experiments: Mean ± SEM, results were analyzed with paired t test. (*p < 0.05; **p < 0.01; ***p < 0.001; ****p < 0.0001).

In an alternative approach, *Rag1* ^-/-^ mice reconstituted with *Irf4* ^+/fl^×*CreER* ^T2^ and *Irf4* ^-/fl^×*CreER* ^T2^ T cells were infected with *C. rodentium*. Five weeks later, after recovery from infection, mice were treated with tamoxifen and further 3 weeks later, CD4^+^ T cells from spleen, mLN and colon were analyzed (**Figure 3A**). With this approach, we tested whether IRF4 was required for the maintenance of Th17 cells. We observed roughly similar populations of *Irf4* ^+/fl^×*CreER* ^T2^ and *Irf4* ^-/fl^×*CreER* ^T2^ CD4^+^ T cells and equal frequencies of GFP^+^ cells in both populations in spleen and mLN (**Figure 3B**; **Supplementary Figure 3**). There was a strong reduction of *Irf4* ^-/fl^×*CreER* ^T2^ CD4^+^ T cells in the colon, and a further reduction of the GFP^+^ subpopulation of these cells. In the *Irf4* ^-/fl^×*CreER* ^T2^ CD4^+^ T cell population, we detected only low frequencies of RORγt^+^ IL-17A^+^ Th17 cells, however, Th17 cells were reliably detectable when gating was limited to either RORγt^+^ or IL-17A^+^ cells (**Figures 3C-E**). Peripheral deletion of an *Irf4* allele did not further reduce the frequencies of Th17 cells in *Irf4* ^+/fl^×*CreER* ^T2^ and *Irf4* ^-/fl^×*CreER* ^T2^ CD4^+^ T cells. Due to the low number of colonic GFP^+^ *Irf4* ^-/-(GFP)^×*CreER* ^T2^ CD4^+^ T cells, we were not able to analyze their RORγt and cytokine expression profile. Under these experimental conditions, *Irf4* ^-/fl^×*CreER* ^T2^ CD4^+^ T cells also had lower frequencies of Th1 cells compared to *Irf4* ^+/fl^ CD4^+^ T cells and Cre-mediated loss of one functional *Irf4* allele further reduced the frequencies of Th1 cells in spleen and mLN (**Figure 3F**). In conclusion, these results confirm the impairment of *Irf4* heterozygous CD4^+^ T cells to differentiate to Th17 cells and further suggest that loss of functional *Irf4* alleles does not prevent maintenance of Th17 cells.

### 3.3 IRF4-deficient mice show an impaired Th2 cell response to *Strongyloides ratti* infection

**Figure 4:**
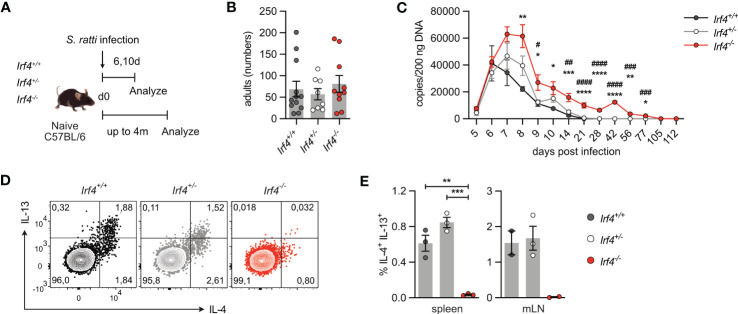
IRF4-deficient mice show impaired Th2 cell response to intestinal *Strongyloides ratti* infection. **(A)** Experimental set up: *Irf4* ^+/+^ *, Irf4* ^+/-^ and *Irf4^-/-^* mice were infected with 2000 *S. ratti* L3 into the hind footpad. Small intestine (SI), mesenteric lymph nodes (mLN) and spleen cells were analyzed after 6 and 10 days post infection. In one group of mice, feces of infected mice were collected for 4 months post infection. **(B)** On day 6 p.i., numbers of *S. ratti* adults in the small intestine of mice were counted. Mean ± SEM of results pooled from 3 independent experiments with 8-12 mice in total. **(C)** Quantitative PCR for *S. ratti*-derived DNA out of feces collected at the indicated time points. Pooled results from two independent experiments with a total of 9-10 mice/group. Mean ± SEM, results were analyzed with one-way ANOVA with Tukey’s multiple comparisons test, * p-values of *Irf4* ^+/+^ versus *Irf4* ^-/-^ mice; ^#^ p-values of *Irf4* ^+/-^ versus *Irf4* ^-/-^ mice. **(D, E)** After 10 days, cells from spleen and mLN were stimulated with PMA and ionomycin and expression of IL-4 and IL-13 by CD4^+^ T cells was examined. **(D)** Representative dot plots for IL-4 and IL-13 of CD4^+^ T cells from mLN of infected mice. **(E)** Representative results for CD4^+^ T cells from spleen and mLN of infected. One of two independent experiments is shown. (* #p < 0.05; ** ##p < 0.01; *** ###p < 0.001; **** ####p < 0.0001).

The role of IRF4 in Th2 cell differentiation and maintenance was analyzed in mice infected with the intestinal nematode *S. ratti* (30, 42). After dermal infection, *S. ratti* larvae migrate to the small intestine (SI) were they mature to reproductive adults. *Irf4* ^+/+^, *Irf4* ^+/-^ and *Irf4* ^-/-^ animals were subcutaneously infected with *S. ratti* larvae into the hind footpad (**Figure 4A**). On day 6 p.i., we detected similar numbers of adult worms in the SI of mice (**Figure 4B**). To monitor the further course of infection*, S. ratti* derived DNA was quantified in the feces of mice as an indicator of egg and first stage larvae (L1) release (**Figure 4C**) (30). Shedding of *S. ratti* L1 and eggs was detected in all mice on day 5 p.i., reached a maximum on day 6-8, and dropped to low levels thereafter. After day 28, all *Irf4* ^+/+^ and *Irf4* ^+/-^ mice had stopped shedding of *S. ratti* L1 and eggs, indicating clearance of infection. In contrast, *Irf4* ^-/-^ mice showed more extensive shedding at the maximum of infection and although shedding subsequently declined, *S. ratti*-DNA was still detected in feces after 11 weeks. Thus, deficiency of IRF4 caused a substantial delay in the control of *S. ratti* infection.

Th2 cells were identified by their cytokine profile and the expression of the transcription factor GATA3. Cells from spleen and mLN were stimulated with PMA and ionomycin and analyzed for cytokine expression (**Figures 4D, E**; **Supplementary Figures 4A, B**). In spleen and mLN of infected *Irf4* ^+/+^ and *Irf4* ^+/-^ mice, we consistently detected IL-4^+^ and IL-13^+^ Th2 cells. In contrast, only marginal frequencies of Th2 cells were detected in tissues of *Irf4* ^-/-^ mice. Spleen and mLN cells of infected *Irf4* ^+/+^, *Irf4* ^+/-^ and *Irf4* ^-/-^ mice were also incubated with *S. ratti* lysate or with anti-CD3 mAb and cytokines in the culture medium were determined (**Supplementary Figures 4C, D**). We detected IL-4, IL-5, IL-13 and IL-10 in the supernatants of *Irf4* ^+/+^ cells both after stimulation with anti-CD3 mAb and *S. ratti* lysates. *Irf4* ^+/-^ cells showed lower production of these cytokines in response to both stimuli when compared to *Irf4* ^+/+^ cells, however, the difference only in some of the assays reached a significant level (p<0.05). *Irf4* ^-/-^ cells produced only marginal levels of these cytokines. In contrast, *Irf4* ^-/-^ cells secreted similar or even higher levels of IFN-γ after anti-CD3 mAb stimulation. Consistent with the marginal production of Th2 cytokines, CD4^+^ T cells from the mLN of *S. ratti* infected *Irf4* ^-/-^ mice also expressed reduced levels of GATA3 (**Supplementary Figure 4E**). In line with their general deficiency in antibody production, *Irf4* ^-/-^ mice failed to generate *S. ratti*-specific IgM, IgG1 and IgG2b (**Supplementary Figure 4F**).

**Figure 5:**
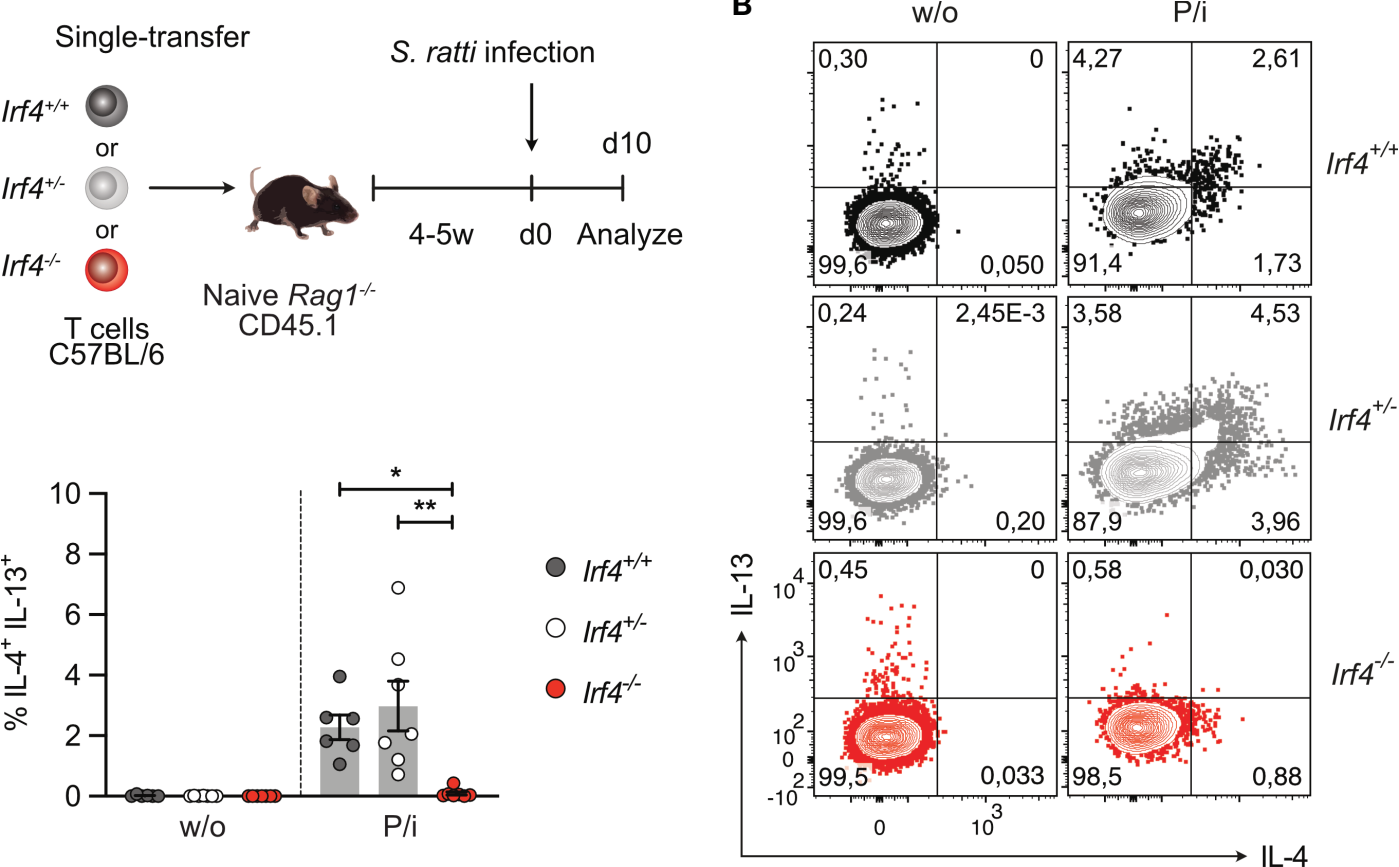
IRF4-deficient T cells fail to generate a Th2 cell response to intestinal *Strongyloides ratti* infection. **(A)** Experimental set up: naive *Rag1* ^−/−^ mice were reconstituted with 4×10^5^ T cells from either *Irf4* ^+/+^ *, Irf4* ^+/-^ or *Irf4* ^-/-^ mice. After 4-5 weeks, recipient mice were infected with 2000 *S. ratti* L3 into the hind footpad. At day 10 post infection, mice were analyzed. **(B, C)** Spleen cells from infected mice were incubated with PMA and ionomycin (P/i) or without (w/o) stimulation and the production of IL-4 and IL-13 by CD4^+^ T cells was determined by flow cytometry. **(B)** Representative dot plots for IL-4 and IL-13 in CD4^+^ T cells. **(C)** Data is representative of two independent experiments with each 6-7 mice/group. Mean ± SEM, results were analyzed with one-way ANOVA with Tukey’s multiple comparisons test. (*p < 0.05; **p < 0.01).

IRF4 is required for ILC2 effector function, namely IL-9 production, and ILC2-derived IL-9 is crucial for the early control (day 6 p.i.) of intestinal *S. ratti* parasite burden (3, 43). Thus, impaired ILC2 effector function might also affect Th2 cell generation in *Irf4* ^-/-^ mice. To proof a T cell intrinsic role of IRF4 in Th2 cell development, *Rag1* ^-/-^ mice were reconstituted with T cells from either *Irf4* ^+/+^, *Irf4* ^+/-^ or *Irf4* ^-/-^ mice (**Figure 5A**). At day 6 after infection, we observed similar shedding of *S. ratti* L1 and eggs in all groups of mice (**Supplementary Figure 5A**). Recipients of *Irf4* ^+/+^ and *Irf4* ^+/-^ cells were able to mount a Th2 cell response, as indicated by intracellular IL-4 and IL-13 staining after polyclonal stimulation of spleen cells (**Figures 5B, C**; **Supplementary Figure 5B**) and IL-4, IL-5, IL-13, and IL-10 secretion after stimulation with anti-CD3 mAb or *S. ratti* lysates (**Supplementary Figure 5C**). In contrast, recipients of *Irf4* ^-/-^ cells were impaired in production of these cytokines. Overall, analysis of reconstituted mice largely reproduced results from *Irf4* ^+/+^, *Irf4* ^+/-^ and *Irf4* ^-/-^ mice indicating that the impaired Th2 cell response was at least in part due to a T cell intrinsic defect in IRF4.

### 3.4 IRF4 is not required for the maintenance of Th2 cells

**Figure 6:**
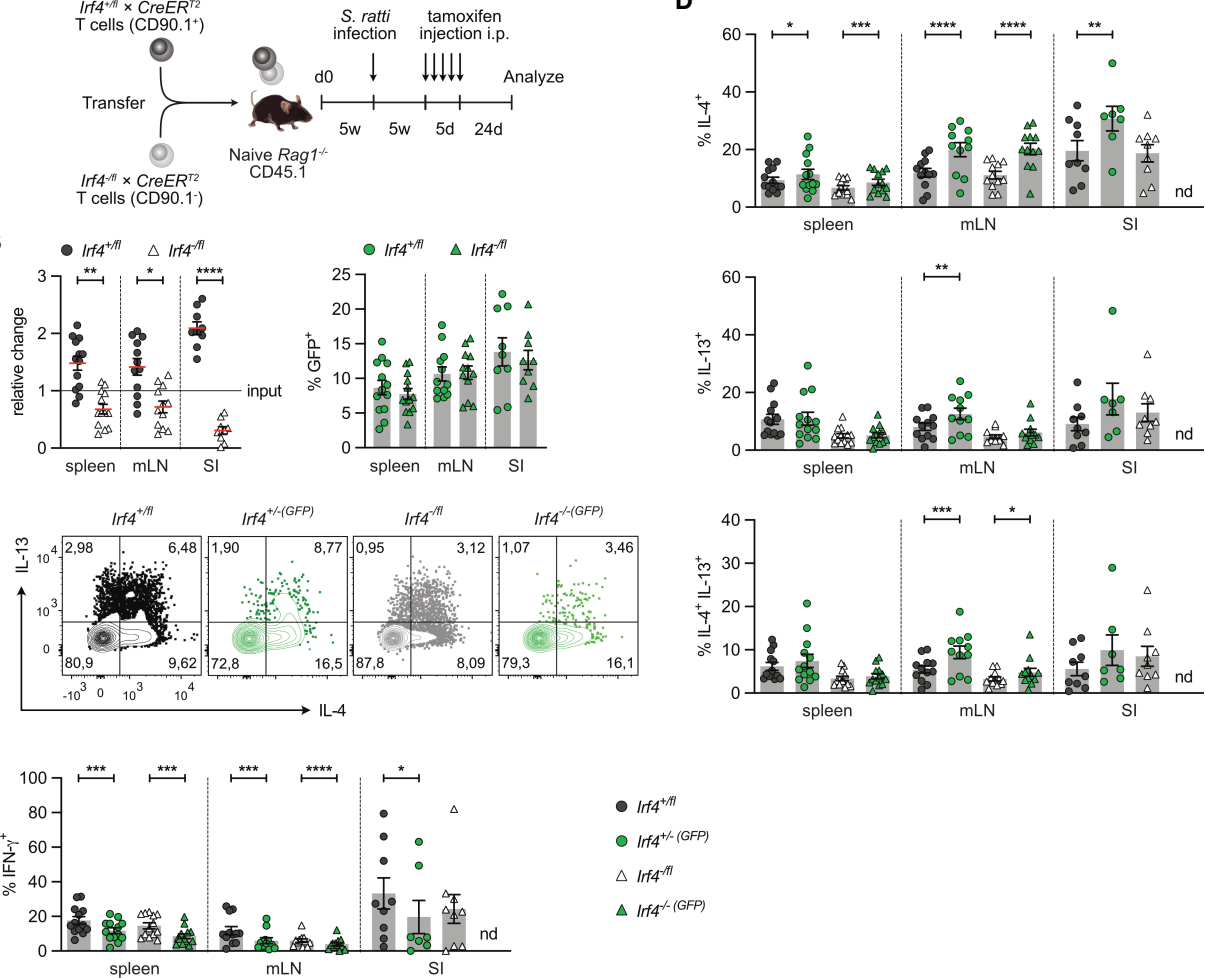
IRF4 is not essential for the maintenance of Th2 cells. **(A)** Experimental set up: naive *Rag1^−/−^* mice were reconstituted with 4×10^5^ T cells from each *Irf4* ^+/fl^×*CreER* ^T2^ (CD90.1^+^) and *Irf4* ^-/fl^×*CreER* ^T2^ (CD90.1^-^) mice. After 4-5 weeks, recipient mice were infected with 2000 *S. ratti* L3 into the hind footpad. Five weeks post infection, mice were treated with tamoxifen for 5 days. Mice were analyzed 24 days post tamoxifen treatment. **(B)** Relative change of the *Irf4* ^+/fl^×*CreER* ^T2^ and *Irf4* ^-/fl^ *×CreER* ^T2^ CD4^+^ T cell population determined by % cells of CD4^+^ T cells at time point of analysis divided by the % cells of transferred CD4^+^ T cells (left). % GFP^+^ cells of *Irf4* ^+/fl^×*CreER* ^T2^ and *Irf4* ^-/fl^×*CreER* ^T2^ CD4^+^ T cells in spleen, mesenteric lymph node (mLN) and small intestine (right). **(C-E)** Cells from spleen, mLN and SI were stimulated with PMA and ionomycin for 4 h, and the frequency of IL-4^+^, IL-13+, IL-4^+^ IL-13^+^ and IFN-γ^+^ CD4^+^ T cells was determined by flow cytometry. **(C)** Representative dot plots for IL-4 and IL-13 in CD4^+^ T cells from mLN. **(B, D, E)** Pooled results from two independent experiments with each 4-8 mice/group. Mean ± SEM, results were analyzed with paired t test. (*p < 0.05; **p < 0.01; ***p < 0.001; ****p < 0.0001).

To analyze, whether IRF4 is required for the maintenance of Th2 cells, we used the transfer model, that allows deletion of *Irf4* in peripheral T cells. *Rag1* ^-/-^ mice reconstituted 1:1 with *Irf4* ^+/fl^×*CreER* ^T2^ and *Irf4* ^-/fl^×*CreER* ^T2^ T cells were s.c. infected with *S. ratti* larvae. Five weeks later, after recovery from infection, mice were treated with tamoxifen and further 3 weeks later, CD4^+^ T cells from spleen, mLN and SI were analyzed (**Figure 6A**). We observed diminished *Irf4* ^-/fl^×*CreER* ^T2^ CD4^+^ T cell accumulation in spleen and mLN, and a strong reduction in the SI (**Figure 6B**; **Supplementary Figure 6**). However, in all tissues there was no significant reduction in frequencies of GFP^+^ cells in both donor cell populations (up to 10-15% of parent donor population). These results confirm that *Irf4* heterozygous mutant CD4^+^ T cells were impaired in accumulating in intestinal tissues and that induced deletion of the remaining functional allele did not further impair this tissue allocation.

In spleen, mLN and SI, we consistently detected IL-4^+^, IL-13^+^, and IL-4^+^IL-13^+^ CD4^+^ T cells in all donor T cell populations except for GFP^+^ *Irf4* ^-/-(GFP)^×*CreER* ^T2^ cells in the SI, of which we were not able to reliably measure cytokine expression due to the very low numbers recovered from tissue (**Figures 6C, D**). In all tissues, heterozygous *Irf4* ^-/fl^×*CreER* ^T2^ CD4^+^ T cells showed similar or slightly lower frequencies of IL-4^+^, IL-13^+^ and IL-4^+^IL-13^+^ Th2 cells when compared to *Irf4* ^+/fl^×*CreER* ^T2^ cells with two functional *Irf4* alleles. Induced deletion of one *Irf4* allele did not decrease the Th2 cytokine expression. We rather observed even higher frequencies of Th2 cytokine positive cells in some of the GFP^+^ populations of both *Irf4* ^+/fl^×*CreER* ^T2^ and *Irf4* ^-/fl^×*CreER* ^T2^ CD4^+^ T cells. In all organs, frequencies of IL-4^+^ T cells were higher in GFP^+^ cells when compared to their parental GFP^-^ cells and frequencies of IL-4^+^IL-13^+^ cells were increased in GFP^+^ cells form the mLN. Thus, under this experimental condition, deletion of one *Irf4* allele rather improved Th2 cell maintenance. Results for Th2 cytokine expression were in contrast with those for IFN-γ expression (**Figure 6E**). Consistent with the results from *C. rodentium* infection, *Irf4* ^-/fl^×*CreER* ^T2^ CD4^+^ T cells had lower frequencies of IFN-γ^+^ cells and induced deletion of one *Irf4* allele reduced the frequencies of IFN-γ^+^ cells in both *Irf4* ^+/fl^×*CreER* ^T2^ and *Irf4* ^-/fl^×*CreER* ^T2^ cells in all analyzed tissues. Overall, these results suggest that that IRF4 is not required for maintenance of Th2 cells.

### 3.5 IRF4-deficient CD4^+^ T cells show reduced expression of intestinal homing receptors and impaired intestinal homing

**Figure 7:**
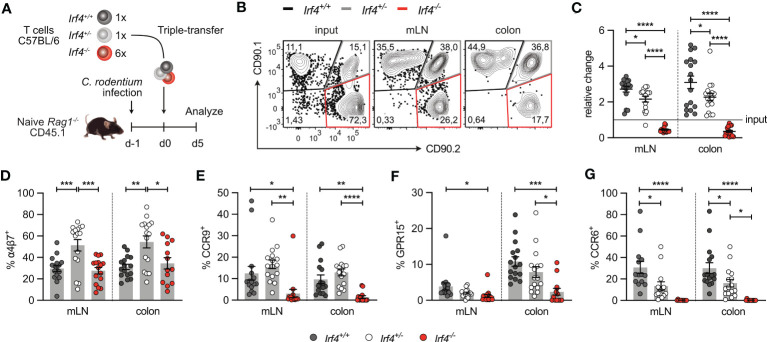
IRF4-deficient CD4^+^ T cells show reduced expression of intestinal homing receptors. **(A)** Experimental set up: naive *Rag1* ^−/−^ mice were infected orally with *C rodentium* and reconstituted one day later with 4×10^5^ T cells of *Irf4^+/+^* (CD90.1^+^) and *Irf4^+/-^* (CD90.1^+^CD90.2^+^), and with different numbers of T cells of *Irf4^-/-^* (CD90.2^+^) mice (either 4×10^5^ or 24×10^5^ cells). 5 days later, mice were analyzed. **(B)** Representative dot plots showing the frequencies of *Irf4^+/+^, Irf4^+/-^* and *Irf4^-/-^* CD4^+^ T cells according to their expression of CD90.1 and CD90.2 at the time point of transfer (input) and after 5 days in mLN and colon. **(C)** Relative change of the *Irf4* ^+/+^, *Irf4* ^+/-^ and *Irf4* ^-/-^ CD4^+^ T cell population determined by % cells of CD4^+^ T cells at time point of analysis divided by the % cells of transferred CD4^+^ T cells. **(D-G)** Percentages of α4β7^+^ **(D)**, CCR9^+^ **(E)**, GPR15^+^ **(F)** and CCR6^+^ **(G)** CD4^+^ T cells in mLN and colon. Pooled results of two independent experiments with each 4 – 12 mice/group. Mean ± SEM, results were analyzed with one-way ANOVA with Tukey’s multiple comparisons test. (*p < 0.05; **p < 0.01; ***p < 0.001; ****p < 0.0001).

In both models of intestinal infection, we observed defective accumulation of *Irf4* heterozygous and particularly *Irf4* homozygous mutant CD4^+^ T cells in SI and colon. To determine the role of IRF4 on their migratory capacity, CD4^+^ T cells from *Irf4* ^+/+^, *Irf4* ^+/-^ and *Irf4* ^-/-^ mice were i.v. co-transferred into *Rag1* ^-/-^ mice that had been infected with *C. rodentium* one day before (**Figure 7A**). Five days after transfer, T cells from mLN and colon were characterized (**Figure 7B**). Compared to the ratio at the transfer, we observed a slight reduction of *Irf4* ^+/-^ cells and a strong reduction of *Irf4* ^-/-^ cells in both tissues, particularly in the colon. (**Figure 7C**). Migration of T cells to intestinal tissues is controlled by the α4β7 integrin and the chemotactic receptors CCR9 and GPR15 (44–48). CD4^+^ T cells in mLN and colon of recipient mice were characterized for expression of these proteins. We also determined expression of CCR6, the hallmark chemokine receptor of Th17 cells (49, 50) (**Figures 7D-G**). In mLN and colon, we observed similar expression of α4β7 on *Irf4* ^+/+^ and *Irf4* ^-/-^ CD4^+^ T cells but increased expression on *Irf4* ^+/-^ T cells. In contrast, CCR9^+^, GPR15^+^ and CCR6^+^ cells were detected on subsets of *Irf4* ^+/+^ and *Irf4* ^+/-^ CD4^+^ T cells but were almost absent on *Irf4* ^-/-^ T cells. Notably, we detected reduced percentages of CCR6^+^ *Irf4* ^+/-^ T cells when compared to *Irf4* ^+/+^ T cells, suggesting that strong CCR6 expression required both *Irf4* alleles.

Largely similar results were obtained when CD4^+^ T cells from tamoxifen-treated *Irf4* ^+/fl^×*CreER* ^T2^ and *Irf4* ^-/fl^×*CreER* ^T2^ mice were transferred into *Rag1^-/-^* mice infected with *C. rodentium* (**Supplementary Figure 7A**). IRF4-deficient T cells (*Irf4* ^-/-(GFP)^×*CreER* ^T2^) showed reduced accumulation in the mLN and particularly in the colon (**Supplementary Figure 7B**), and under these conditions, IRF4-deficient CD4^+^ T cells in mLN and colon were impaired in the expression of α4β7, CCR9, GPR15 and CCR6 (**Supplementary Figures 7C-F**). Collectively, these results demonstrate that IRF4 controls expression of intestinal homing receptors on CD4^+^ T cells and is thus required for accumulation of these cells in the intestinal mucosa.

**Figure 8:**
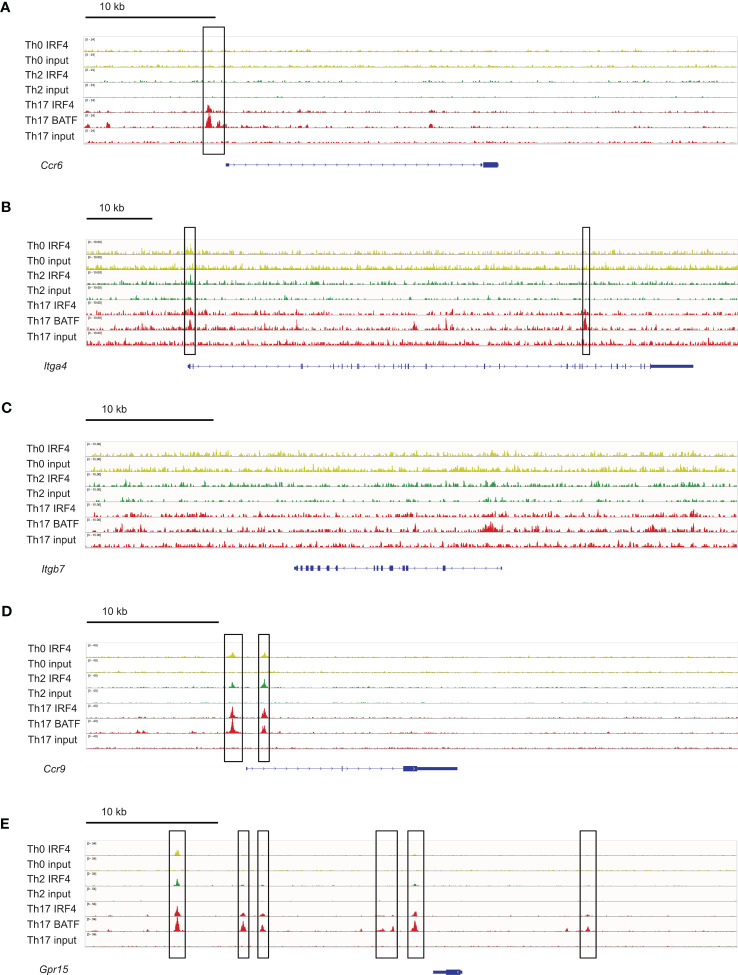
IRF4 binding sites in genes coding for intestinal homing receptors. ChIP-seq data sets for *in vitro* differentiated Th0, Th2 and Th17 cells (4) were analyzed for IRF4 binding sites in the gene loci for *Ccr6* **(A)**, *Itga4* **(B)**, *Itgb7* **(C)**, *Ccr9* **(D)** and *Gpr15* **(E)**. IRF4 binding sites are marked with a frame.

To determine if IRF4 can directly control the expression of these homing receptors, we analyzed a chromatin immunoprecipitation sequencing (ChIP-seq) data set from Glasmacher and colleagues (4), in which IRF4 DNA complexes were precipitated from *in vitro* differentiated murine Th0, Th2 and Th17 cells. As expected, we detected IRF4 binding 5’ of the *Ccr6* gene in Th17 cells but not in Th0 or Th2 cells (**Figure 8A**). IRF4 binding was also detected in the *Itga4* gene locus, with strong signals in Th17 cells and week signals in Th0 and Th2 cells (**Figure 8B**). In contrast, in the *Itgb7* gene locus, IRF4 binding was at background level in all T cell subsets (**Figure 8C**). For *Ccr9*, IRF4 binding was detected in the 5’ region of the gene locus of all Th cell subsets (**Figure 8D**) and for *Gpr15* several binding sites were observed upstream of the gene, with particular strong signals in Th17 cells (**Figure 8E**). Overall these results suggest that IRF4 can directly control the expression of CCR6 and of the intestinal homing receptors.

## 4 Discussion

Following infection with *C. rodentium* or *S. ratti*, *Irf4* ^-/-^ mice failed to control infection, resulting in systemic dissemination of *C. rodentium* and delayed clearance of *S. ratti*, respectively. Impaired pathogen control was associated with a defective development of Th17 and Th2 cells and limited intestinal accumulation of CD4^+^ T cells. These results are consistent with the general deficiency of *Irf4* ^-/-^ mice to mount effector T cell responses, including Th17 and Th2 cell responses, and a limited ability of *Irf4* ^-/-^ T cells to migrate to intestinal tissues (1, 13, 15).

*Irf4* ^-/-^ mice showed reduced accumulation of CD4^+^ T cells in the colon, both under homeostatic conditions and after infections. This result is consistent with a study from Mudter et al. (51) which reports impaired migration of *Irf4^-/-^* T cells to the intestine in a colitis model. In our T cell transfer assays, deficiency of one or both *Irf4* alleles caused reduced CD4^+^ T cell accumulation in colon and small intestine, indicating that the defect is T cell intrinsic and that under competitive conditions, lower expression levels of IRF4, due to loss of one allele, prevent effective intestinal T cell accumulation. However, induced loss of *Irf4* alleles after the transfer of T cells had only limited effects on the persistence of cells in the colon and no effect in the small intestine. Thus, IRF4 appears to be important for migration of CD4^+^ T cells into the intestine but has only a minor role for their intestinal persistence. The impaired migration of *Irf4* heterozygous T cells to the intestine and possible to other peripheral tissues could also explain the detection of several cases of Whipple's disease in an *Irf4* haploinsufficient kindred (52). Whipple’s disease is caused by the Gram-positive actinomycete *Tropheryma whipplei* and is characterized by mainly intestinal but also extra-intestinal manifestations. *T. whipplei* is widespread and infection most likely occurs via oral uptake. However, typically only a very small minority of infected individuals develops Whipple’s disease (53, 54). Analysis of gene loci of intestinal homing receptors revealed potential IRF4 binding sites in *Ccr9*, *Gpr15* and *Itga4* suggesting a direct regulation of these genes by IRF4. As a likely consequence, *Irf4* ^-/-^ CD4^+^ T cells fail to express CCR9 and GPR15 which could be responsible for the defective intestinal accumulation. For GPR15, we also observed a trend for lower expression in *Irf4* ^+/-^ CD4^+^ T cells which might explain their reduced intestinal migration under competitive conditions. Differences in expression of intestinal homing receptors were more pronounced in the inducible deletion model. We observed reduced expression of α4β7, CCR9 and GPR15 in heterozygous *Irf4* ^-/fl^ CD4^+^ T cells and almost complete lack of expression of α4β7 and CCR9 after deletion of the 2^nd^ *Irf4* allele. Overall, these results indicate that the expression levels of IRF4 in CD4^+^ T cells determine their capacity to migrate to the intestine. However, we could show that IRF4 is less important for their intestinal persistence.

*Irf4* ^-/-^ mice with a global deficiency failed to mount Th17 and Th2 cell responses to *C. rodentium* and *S. ratti* infection, respectively. *Irf4* ^+/-^ mice were not impaired in the development of Th17 or Th2 cell responses, rather, we observed a trend to even higher frequencies of cytokine secreting cells after *in vitro* stimulation of cells. The situation was different after competitive T cell transfer, where heterozygous *Irf4* ^-/fl^×*CreER* ^T2^ T cells showed reduced Th17 and Th2 cell responses when compared to co-transferred *Irf4* ^+/fl^×*CreER* ^T2^ T cells with two functional *Irf4* alleles. The observed effect could be due to competition of *Irf4* heterozygous T cells with simultaneously transferred wt T cells. Thus, lower IRF4 levels are of disadvantage for the differentiation of Th17 and Th2 cells which is in line with the dose effect for IRF4 observed for the intestinal migration of CD4^+^ T cells but also with published studies for CD8^+^ and CD4^+^ Th1 cells (22–25). Induced deletion of one *Irf4* allele in *Irf4* ^+/fl^×*CreER* ^T2^ CD4^+^ T cells after transfer did not impair the cytokine production upon restimulation. For some tissue, we observed even slightly higher frequencies of IL-4^+^, IL-13^+^ or IL-17^+^ CD4^+^ T cells. For CD8^+^ T cells, is has been shown that during chronic infection low IRF4 levels can limit development of T cell exhaustion and that CAR T cells need to limit the function of AICE binding complexes to remain functional (26). Similarly, induced loss of one *Irf4* allele could support maintenance of responsiveness of Th17 and Th2 cells. Deletion of the second *Irf4* allele in *Irf4* ^-/fl^×*CreER* ^T2^ CD4^+^ T cells after recovery from *C. rodentium* or *S. ratti* infection had no or only limited effects on the frequencies of Th17 or Th2 cells in spleen and mLN suggesting that IRF4 is not essential for their survival in these tissues and for their capacity to secrete Th17 and Th2 cytokines after re-stimulation. Thus, at least for IL-17A as well as for IL-4 and IL-13, IRF4 is not required to maintain the accessibility of this cytokine loci and for the induction of cytokine expression upon restimulation.

Interestingly, we still observed IL-17A production and low RORγt expression in *Irf4* ^-/-(GFP)^×*CreER* ^T2^ CD4^+^ T cells when the second allele was deleted in recipient mice prior to the *C. rodentium* infection, which contrasts CD4^+^ T cells from *Irf4* ^-/-^ mice and would indicate that IRF4 is not essential at this time point of T cell differentiation. Furthermore, deletion of an *Irf4* allele, in *Irf4* ^+/fl^×*CreER* ^T2^ T cells prior to transfer did not diminish Th17 cell differentiation of these cells. We currently have no explanation for this discrepancy. The *Irf4* genotype of T cells and of the environment during thymic maturation could affect their ability for Th17 cell differentiation. Low level of IRF4 expression in peripheral CD4^+^ T cells could also be sufficient to induce a predetermined state that allows some Th17 cell differentiation in the subsequent absence of IRF4. For *Irf4* ^+/fl^×*CreER* ^T2^ CD4^+^ T cells, we can also not exclude that some T cells have differentiated to Th17 cells prior to transfer, e.g., in response to the intestinal flora, and after deletion of the *Irf4* allele are still able to expand in response to the *C. rodentium* induced inflammation.

With the protocol of tamoxifen application over 5 days, we observe a deletion of one floxed *Irf4* allele in 5-15% of CD4^+^ T cells as indicated by the induction of GFP [**Figure 2A** and (20)]. GFP expression closely correlates with reduction of IRF4 expression (**Supplementary Figure 2**) and with the deletion of exon 2 (25) indicating that GFP expression does not underreport *Irf4* deletion. Due to the low efficacy, we abstained from using T cells from *Irf4* ^fl/fl^×*CreER* ^T2^ mice since the efficacy of deletion of both alleles is far lower (<2%) and cannot be distinguished from deletion of only one allele by the GFP expression. As a consequence of the low efficacy, only a very low number of *Irf4* ^-/- (GFP)^×*CreER* ^T2^ CD4^+^ T cells could be recovered from colon and small intestine (usually less than 20 cells per sample). Therefore, we provide results on the frequencies of these rare cells in small and large intestine but refrain from showing results on their cytokine or RORγt expression profile. However, in other tissues the relative low efficacy is even of advantage as long as cells with and without Cre-induced deletion can be reliably distinguished. The low rate of deletion allows the analysis of the altered T cells in an otherwise unchanged environment with a majority of unaltered T cells and thus in the absence of secondary effects due to a generally impaired T cell response.

After induced deletion of the remaining floxed *Irf4* allele, in *Irf4* ^-/-(GFP)^×*CreER* ^T2^ CD4^+^ T cells might still be persisting *Irf4* mRNA or IRF4 protein that could mask the knockout phenotype of the cells. However, in all our assays, we waited at least 11-12 days, in most cases >20 days after tamoxifen treatment before the analyses of cells, and as mentioned before, expression of IRF4 protein correlated with the phenotype. In addition, for IFN-γ or IFN-γ and TNF-α, we observed consistently a reduction of cytokine producing cells after induced deletion of the second *Irf4* allele, which argues against an activity of persisting *Irf4* mRNA or IRF4 protein. Of note, we have currently no explanation for the dissimilar effect of *Irf4* deletion on different cytokines.

For Th17 and Th2 cells, induced deletion of one *Irf4* allele after recovery of infection had no effect on their cytokine response after T cell stimulation and did not impair their persistence, at least within the analyzed time period. However, this does not indicate that these cells can mount a regular secondary T cell response. During primary T cell responses, IRF4 is required for fundamental processes such as adaptation of the cellular metabolism to the requirements of proliferating and effector protein production. For CD8^+^ T cells, *Irf4* deletion after recovery of infection completely prevented expansion of the memory T cell population following re-infection (25) and it is very likely that this holds true for secondary responses of Th17 and Th2 cells as well.

## Data availability statement

The original contributions presented in the study are included in the article/**Supplementary Material**. Further inquiries can be directed to the corresponding authors.

## Ethics statement

The animal study was reviewed and approved by Local committee for animal experiments of the City of Hamburg (registration numbers: N017/2017, N055/2019 N068/2021, N150/2021).

## Author contributions

CS, FR, MB and H-WM designed research. CS, AH, DR, LCV, JS, TB, NCL, and FR performed research. FK-N and SH contributed new reagents/analytic tools. CS, FR, MS, MB and H-WM analyzed data. CS, FR and H-WM wrote the paper. All authors contributed to the article and approved the submitted version.

## References

[1] Huber M Lohoff M. IRF4 at the crossroads of effector T-cell fate decision. Eur J Immunol (2014) 44(7):1886–95. doi: 10.1002/eji.201344279 24782159

[2] Lohoff M Mak TW. Roles of interferon-regulatory factors in T-helper-cell differentiation. Nat Rev Immunol (2005) 5(2):125–35. doi: 10.1038/nri1552 15688040

[3] Mohapatra A Van Dyken SJ Schneider C Nussbaum JC Liang HE Locksley RM. Group 2 innate lymphoid cells utilize the IRF4-IL-9 module to coordinate epithelial cell maintenance of lung homeostasis. Mucosal Immunol (2016) 9(1):275–86. doi: 10.1038/mi.2015.59 PMC4698110 26129648

[4] Glasmacher E Agrawal S Chang AB Murphy TL Zeng W Vander Lugt B. A genomic regulatory element that directs assembly and function of immune-specific AP-1-IRF complexes. Science (2012) 338(6109):975–80. doi: 10.1126/science.1228309 PMC5789805 22983707

[5] Li P Spolski R Liao W Wang L Murphy TL Murphy KM. BATF-JUN is critical for IRF4-mediated transcription in T cells. Nature (2012) 490(7421):543–6. doi: 10.1038/nature11530 PMC3537508 22992523

[6] Iwata A Durai V Tussiwand R Briseno CG Wu X Grajales-Reyes GE. Quality of TCR signaling determined by differential affinities of enhancers for the composite BATF-IRF4 transcription factor complex. Nat Immunol (2017) 18(5):563–72. doi: 10.1038/ni.3714 PMC5401770 28346410

[7] Murphy TL Tussiwand R Murphy KM. Specificity through cooperation: BATF-IRF interactions control immune-regulatory networks. Nat Rev Immunol (2013) 13(7):499–509. doi: 10.1038/nri3470 23787991

[8] Ciofani M Madar A Galan C Sellars M Mace K Pauli F. A validated regulatory network for Th17 cell specification. Cell (2012) 151(2):289–303. doi: 10.1016/j.cell.2012.09.016 23021777 PMC3503487

[9] Man K Kallies A. Synchronizing transcriptional control of T cell metabolism and function. Nat Rev Immunol (2015) 15(9):574–84. doi: 10.1038/nri3874 26272293

[10] Man K Miasari M Shi W Xin A Henstridge DC Preston S. The transcription factor IRF4 is essential for TCR affinity-mediated metabolic programming and clonal expansion of T cells. Nat Immunol (2013) 14(11):1155–65. doi: 10.1038/ni.2710 24056747

[11] Rengarajan J Mowen KA Mcbride KD Smith ED Singh H Glimcher LH. Interferon regulatory factor 4 (IRF4) interacts with NFATc2 to modulate interleukin 4 gene expression. J Exp Med (2002) 195(8):1003–12. doi: 10.1084/jem.20011128 PMC2193700 11956291

[12] Cretney E Xin A Shi W Minnich M Masson F Miasari M. The transcription factors blimp-1 and IRF4 jointly control the differentiation and function of effector regulatory T cells. Nat Immunol (2011) 12(4):304–11. doi: 10.1038/ni.2006 21378976

[13] Brüstle A Heink S Huber M Rosenplanter C Stadelmann C Yu P. The development of inflammatory T(H)-17 cells requires interferon-regulatory factor 4. Nat Immunol (2007) 8(9):958–66. doi: 10.1038/ni1500 17676043

[14] Krishnamoorthy V Kannanganat S Maienschein-Cline M Cook SL Chen J Bahroos N. The IRF4 gene regulatory module functions as a read-write integrator to dynamically coordinate T helper cell fate. Immunity (2017) 47(3):481–97 e7. doi: 10.1016/j.immuni.2017.09.001 28930660 PMC5661949

[15] Lohoff M Mittrücker HW Prechtl S Bischof S Sommer F Kock S. Dysregulated T helper cell differentiation in the absence of interferon regulatory factor 4. Proc Natl Acad Sci U.S.A. (2002) 99(18):11808–12. doi: 10.1073/pnas.182425099 PMC129350 12189207

[16] Lohoff M Ferrick D Mittrücker H-W Duncan GS Bischof S Röllinghoff M. Interferon regulatory factor-1 is required for a T helper 1 immune response. In Vivo Immun (1997) 6(6):681–9. doi: 10.1016/s1074-7613(00)80444-6 9208841

[17] Tominaga N Ohkusu-Tsukada K Udono H Abe R Matsuyama T Yui K. Development of Th1 and not Th2 immune responses in mice lacking IFN-regulatory factor-4. Int Immunol (2003) 15(1):1–10. doi: 10.1093/intimm/dxg001 12502720

[18] Raczkowski F Ritter J Heesch K Schumacher V Guralnik A Hocker L. The transcription factor interferon regulatory factor 4 is required for the generation of protective effector CD8+ T cells. Proc Natl Acad Sci U.S.A. (2013) 110(37):15019–24. doi: 10.1073/pnas.1309378110 PMC3773801 23980171

[19] Bravo Garcia-Morato M Aracil Santos FJ Briones AC Blazquez Moreno A Del Pozo Mate A Dominguez-Soto A. New human combined immunodeficiency caused by interferon regulatory factor 4 (IRF4) deficiency inherited by uniparental isodisomy. J Allergy Clin Immunol (2018) 141(5):1924–7 e18. doi: 10.1016/j.jaci.2017.12.995 29408330

[20] Klein U Casola S Cattoretti G Shen Q Lia M Mo T. Transcription factor IRF4 controls plasma cell differentiation and class-switch recombination. Nat Immunol (2006) 7(7):773–82. doi: 10.1038/ni1357 16767092

[21] Mittrücker HW Matsuyama T Grossman A Kundig TM Potter J Shahinian A. Requirement for the transcription factor LSIRF/IRF4 for mature b and T lymphocyte function. Science (1997) 275(5299):540–3. doi: 10.1126/science.275.5299.540 8999800

[22] Nayar R Schutten E Bautista B Daniels K Prince AL Enos M. Graded levels of IRF4 regulate CD8+ T cell differentiation and expansion, but not attrition, in response to acute virus infection. J Immunol (2014) 192(12):5881–93. doi: 10.4049/jimmunol.1303187 PMC4080788 24835398

[23] Nayar R Schutten E Jangalwe S Durost PA Kenney LL Conley JM. IRF4 regulates the ratio of T-bet to eomesodermin in CD8+ T cells responding to persistent LCMV infection. PloS One (2015) 10(12):e0144826. doi: 10.1371/journal.pone.0144826 26714260 PMC4699851

[24] Man K Gabriel SS Liao Y Gloury R Preston S Henstridge DC. Transcription factor IRF4 promotes CD8(+) T cell exhaustion and limits the development of memory-like T cells during chronic infection. Immunity (2017) 47(6):1129–41 e5. doi: 10.1016/j.immuni.2017.11.021 29246443

[25] Harberts A Schmidt C Schmid J Reimers D Koch-Nolte F Mittrücker H-W. Interferon regulatory factor 4 controls effector functions of CD8+ memory T cells. Proc Natl Acad Sci U.S.A. (2021) 118(16):e2014553118. doi: 10.1073/pnas.2014553118 33859042 PMC8072204

[26] Seo H Gonzalez-Avalos E Zhang W Ramchandani P Yang C Lio CJ. BATF and IRF4 cooperate to counter exhaustion in tumor-infiltrating CAR T cells. Nat Immunol (2021) 22(8):983–95. doi: 10.1038/s41590-021-00964-8 PMC8319109 34282330

[27] Mombaerts P Iacomini J Johnson RS Herrup K Tonegawa S Papaioannou VE. RAG-1-deficient mice have no mature b and T lymphocytes. Cell (1992) 68(5):869–77. doi: 10.1016/0092-8674(92)90030-g 1547488

[28] Hameyer D Loonstra A Eshkind L Schmitt S Antunes C Groen A. Toxicity of ligand-dependent Cre recombinases and generation of a conditional Cre deleter mouse allowing mosaic recombination in peripheral tissues. Physiol Genomics (2007) 31(1):32–41. doi: 10.1152/physiolgenomics.00019.2007 17456738

[29] Viney ME Lok JB. Strongyloides spp. WormBook, ed. The C. elegans Research Community, WormBook. (2007). doi: 10.1895/wormbook.1.141.1

[30] Eschbach ML Klemm U Kolbaum J Blankenhaus B Brattig N Breloer M. Strongyloides ratti infection induces transient nematode-specific Th2 response and reciprocal suppression of IFN-gamma production in mice. Parasite Immunol (2010) 32(5):370–83. doi: 10.1111/j.1365-3024.2010.01199.x 20500666

[31] Rissiek B Lukowiak M Raczkowski F Magnus T Mittrücker HW Koch-Nolte F. *In vivo* blockade of murine ARTC2.2 during cell preparation preserves the vitality and function of liver tissue-resident memory T cells. Front Immunol (2018) 9:1580. doi: 10.3389/fimmu.2018.01580 30038627 PMC6046629

[32] Anderson KG Mayer-Barber K Sung H Beura L James BR Taylor JJ. Intravascular staining for discrimination of vascular and tissue leukocytes. Nat Protoc (2014) 9(1):209–22. doi: 10.1038/nprot.2014.005 PMC4428344 24385150

[33] Chen S Zhou Y Chen Y Gu J. Fastp: an ultra-fast all-in-one FASTQ preprocessor. Bioinformatics (2018) 34(17):i884–i90. doi: 10.1093/bioinformatics/bty560 PMC6129281 30423086

[34] Langmead B Salzberg SL. Fast gapped-read alignment with bowtie 2. Nat Methods (2012) 9(4):357–9. doi: 10.1038/nmeth.1923 PMC3322381 22388286

[35] Danecek P Bonfield JK Liddle J Marshall J Ohan V Pollard MO. Twelve years of SAMtools and BCFtools. Gigascience (2021) 10(2):giab008. doi: 10.1093/gigascience/giab008 33590861 PMC7931819

[36] Zhang Y Liu T Meyer CA Eeckhoute J Johnson DS Bernstein BE. Model-based analysis of ChIP-seq (MACS). Genome Biol (2008) 9(9):R137. doi: 10.1186/gb-2008-9-9-r137 18798982 PMC2592715

[37] Langmead B Trapnell C Pop M Salzberg SL. Ultrafast and memory-efficient alignment of short DNA sequences to the human genome. Genome Biol (2009) 10(3):R25. doi: 10.1186/gb-2009-10-3-r25 19261174 PMC2690996

[38] Quinlan AR Hall IM. BEDTools: a flexible suite of utilities for comparing genomic features. Bioinformatics (2010) 26(6):841–2. doi: 10.1093/bioinformatics/btq033 PMC2832824 20110278

[39] Robinson JT Thorvaldsdottir H Winckler W Guttman M Lander ES Getz G. Integrative genomics viewer. Nat Biotechnol (2011) 29(1):24–6. doi: 10.1038/nbt.1754 PMC3346182 21221095

[40] Bishu S Hou G El Zaatari M Bishu SR Popke D Zhang M. Citrobacter rodentium induces tissue-resident memory CD4(+) T cells. Infect Immun (2019) 87(7):e00295–19. doi: 10.1128/IAI.00295-19 PMC6589064 31061145

[41] Collins JW Keeney KM Crepin VF Rathinam VA Fitzgerald KA Finlay BB. Citrobacter rodentium: infection, inflammation and the microbiota. Nat Rev Microbiol (2014) 12(9):612–23. doi: 10.1038/nrmicro3315 25088150

[42] Breloer M Abraham D. Strongyloides infection in rodents: immune response and immune regulation. Parasitology (2017) 144(3):295–315. doi: 10.1017/S0031182016000111 26905057

[43] Meiners J Reitz M Rudiger N Turner JE Heepmann L Rudolf L. IL-33 facilitates rapid expulsion of the parasitic nematode strongyloides ratti from the intestine via ILC2- and IL-9-driven mast cell activation. PloS Pathog (2020) 16(12):e1009121. doi: 10.1371/journal.ppat.1009121 33351862 PMC7787685

[44] Berlin C Berg EL Briskin MJ Andrew DP Kilshaw PJ Holzmann B. α4β7 integrin mediates lymphocyte binding to the mucosal vascular addressin MAdCAM-1. Cell (1993) 74(1):185–95. doi: 10.1016/0092-8674(93)90305-a 7687523

[45] Kantele A Zivny J Hakkinen M Elson CO Mestecky J. Differential homing commitments of antigen-specific T cells after oral or parenteral immunization in humans. J Immunol (1999) 162(9):5173–7. doi: 10.4049/jimmunol.162.9.5173 10227989

[46] Kim SV Xiang WV Kwak C Yang Y Lin XW Ota M. GPR15-mediated homing controls immune homeostasis in the large intestine mucosa. Science (2013) 340(6139):1456–9. doi: 10.1126/science.1237013 PMC3762262 23661644

[47] Nguyen LP Pan J Dinh TT Hadeiba H O'hara E 3rd Ebtikar A. Role and species-specific expression of colon T cell homing receptor GPR15 in colitis. Nat Immunol (2015) 16(2):207–13. doi: 10.1038/ni.3079 PMC4338558 25531831

[48] Habtezion A Nguyen LP Hadeiba H Butcher EC. Leukocyte trafficking to the small intestine and colon. Gastroenterology (2016) 150(2):340–54. doi: 10.1053/j.gastro.2015.10.046 PMC4758453 26551552

[49] Wang C Kang SG Lee J Sun Z Kim CH. The roles of CCR6 in migration of Th17 cells and regulation of effector T-cell balance in the gut. Mucosal Immunol (2009) 2(2):173–83. doi: 10.1038/mi.2008.84 PMC2709747 19129757

[50] Turner JE Paust HJ Steinmetz OM Peters A Riedel JH Erhardt A. CCR6 recruits regulatory T cells and Th17 cells to the kidney in glomerulonephritis. J Am Soc Nephrol (2010) 21(6):974–85. doi: 10.1681/ASN.2009070741 PMC2900961 20299360

[51] Mudter J Amoussina L Schenk M Yu J Brustle A Weigmann B. The transcription factor IFN regulatory factor-4 controls experimental colitis in mice via T cell-derived IL-6. J Clin Invest (2008) 118(7):2415–26. doi: 10.1172/JCI33227 PMC2413182 18535667

[52] Guerin A Kerner G Marr N Markle JG Fenollar F Wong N. IRF4 haploinsufficiency in a family with whipple's disease. Elife (2018) 7:e32340. doi: 10.7554/eLife.32340 29537367 PMC5915175

[53] Marth T Moos V Muller C Biagi F Schneider T. Tropheryma whipplei infection and whipple's disease. Lancet Infect Dis (2016) 16(3):e13–22. doi: 10.1016/S1473-3099(15)00537-X 26856775

[54] Boumaza A Ben Azzouz E Arrindell J Lepidi H Mezouar S Desnues B. Whipple's disease and tropheryma whipplei infections: from bench to bedside. Lancet Infect Dis (2022) 22(10):e280–e91. doi: 10.1016/S1473-3099(22)00128-1 35427488

