# Supplementary material for: IRF4 is required for migration of CD4+ T cells to the intestine but not for Th2 and Th17 cell maintenance
Source: Front Immunol. 2023 Jul 3;14:1182502. doi: 10.3389/fimmu.2023.1182502 (PMC10352983; doi:10.3389/fimmu.2023.1182502)
Supplement: Supplementary file 1 [file DataSheet_1.pdf]

Supplementary Material

IRF4 is required for migration of CD4$^+$ T cells to the intestine but not for Th2 and Th17 cell maintenance

Constantin Schmidt[1, 2], Aenne Harberts[1, 2], Daniel Reimers[1], Tabea Bertram[1], Leonie Caroline Voß[1], Joanna Schmid[1], Niels Christian Lory[1], Michael Spohn[3, 4, 5], Friedrich Koch-Nolte[1], Samuel Huber[2], Friederike Raczkowski[1]†, Minka Breloer[6, 7]† and Hans-Willi Mittrücker[1]†

[1]Department for Immunology, University Medical Center Hamburg-Eppendorf, Hamburg, Germany, [2]I. Department of Medicine, University Medical Center Hamburg-Eppendorf, Hamburg, Germany, [3]Clinic of Pediatric Hematology and Oncology, University Medical Center Hamburg-Eppendorf, Hamburg, Germany, [4]Research Institute Children's Cancer Center Hamburg, Hamburg, Germany, [5]Bioinformatics Core Unit, University Medical Center Hamburg-Eppendorf, Hamburg, Germany, [6]Section for Molecular Biology and Immunology, Bernhard Nocht Institute for Tropical Medicine, Hamburg, Germany, [7]Department for Biology, University Hamburg, Hamburg, Germany

† These authors contributed equally to this work and share last authorship.

*** Correspondence:**
Corresponding Authors
con.schmidt@uke.de, h.mittruecker@uke.de

Suppl. Figure 1

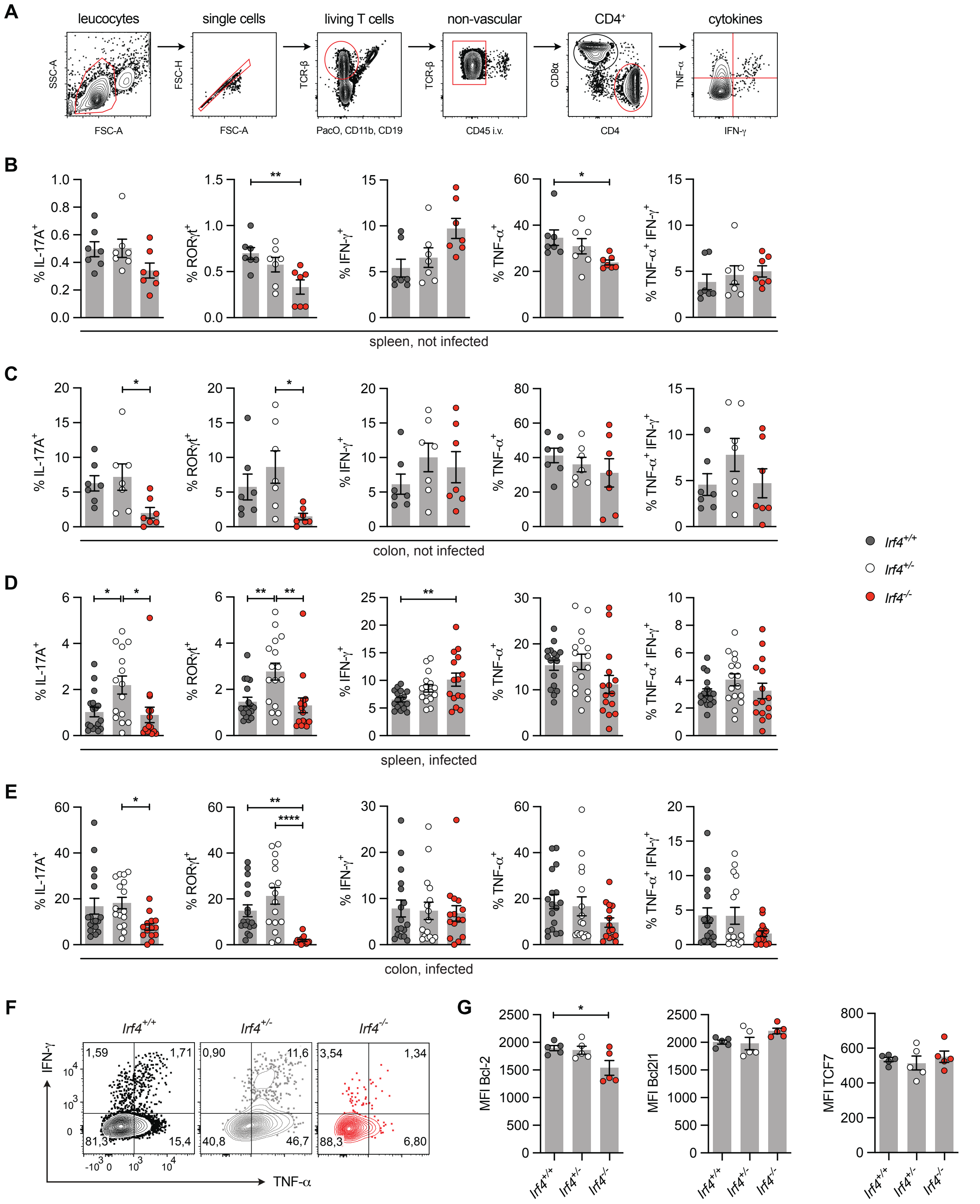

Suppl. Figure 1. IRF4-deficient mice show impaired Th17 cell response to intestinal *Citrobacter rodentium* infection.
(A) Gating strategy for cytokines in single living $CD11b^-$ $CD19^-$ CD45i.v.$^-$ (non-vascular) βTCR^+ $CD4^+$ T cells. To label vascular cells, mice were i.v. injected with fluorochrome-conjugated anti-CD45 mAb 3-5 min before harvesting of tissues. Isolated cells were stimulated for 4h with PMA and ionomycin to induce cytokine expression. (**B**-**E**) Mice were treated as described in Fig. 1A. Percentages of IL-17A$^+$, RORγt$^+$, TNF-α$^+$ and IFN-γ$^+$ $CD4^+$ T cells after 4 hours of stimulation with PMA and ionomycin from spleen (**B**, **D**) and colon (**C**, **E**) of naive (**B**, **C**) and infected (**D**, **E**) *Irf4*$^{+/+}$, *Irf4*$^{+/-}$ and *Irf4*$^{-/-}$ mice. Pooled results of 2 independent experiments in naive groups and 3 independent experiments in infected groups. (**F**) Representative FACS plots of TNF-α and IFN-γ -producing $CD4^+$ T cells from colon of infected mice after stimulation with PMA and ionomycin. (**G**) Expression of the anti-apoptotic proteins Bcl2, Bcl-2l1 and of the transcription factor TCF-7 as determined by intracellular staining and flow cytometry in $CD4^+$ T cells from spleens of naïve mice (MFI, mean fluorescence intensity). Results from one experiment are shown. (**B-E, G**) Mean ± SEM, results were analyzed with one-way ANOVA with Tukey's multiple comparisons posttest. (*$p < 0.05$; **$p < 0.01$; ****$p < 0.0001$)

Suppl. Figure 2

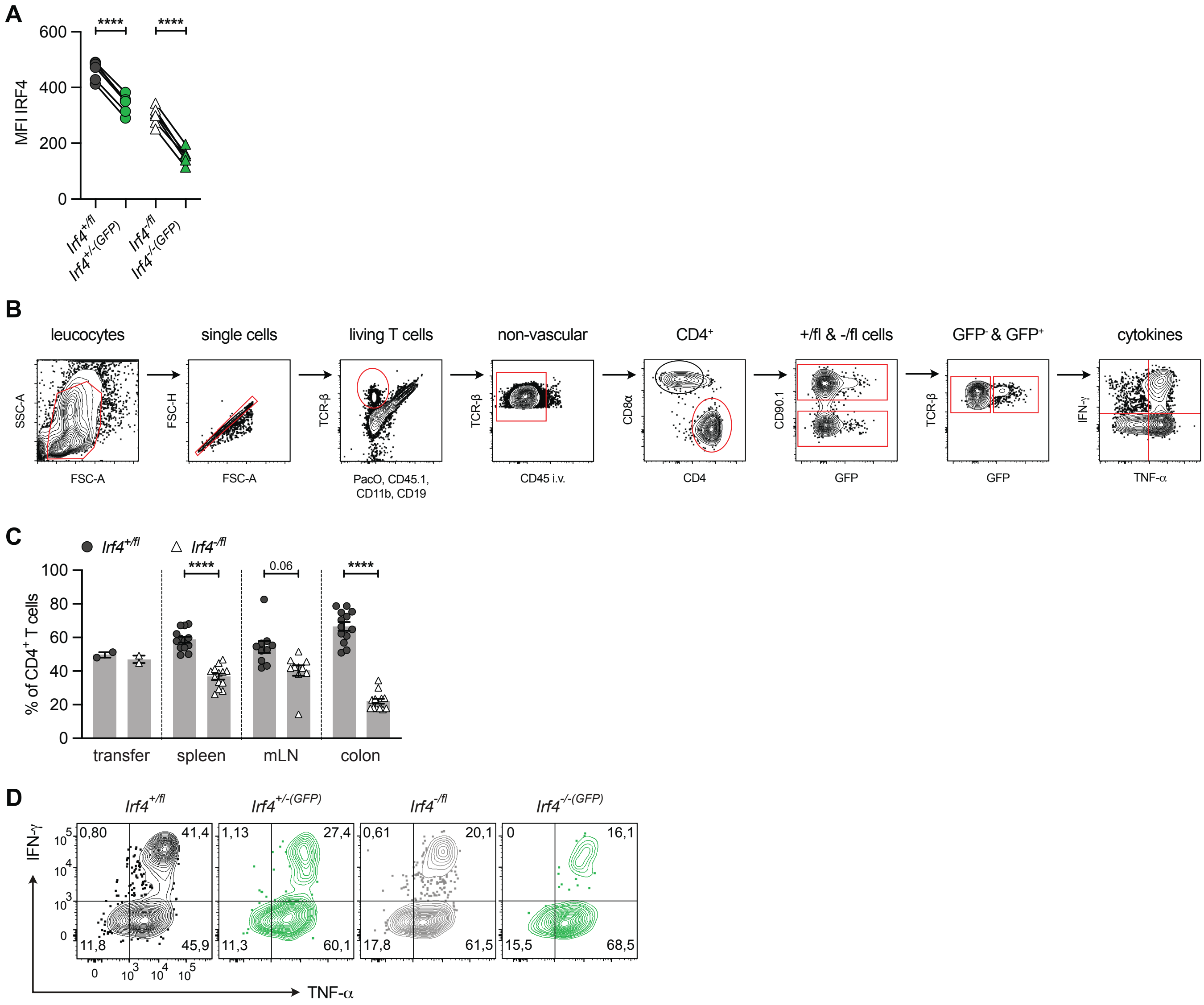

Suppl. Figure 2. IRF4 dose dependent impairment of Th17 cell differentiation.
(**A**) IRF4 expression of *Irf4*$^{+/fl}$×*CreER*^{T2}, *Irf4*$^{+/-(GFP)}$×*CreER*^{T2}, *Irf4*$^{-/fl}$×*CreER*^{T2} and *Irf4*$^{-/-(GFP)}$×*CreER*^{T2} CD4^{+} T cells. Spleen cells were isolated from *Rag1*$^{-/-}$ recipient mice as described in Figure 2B. Cells were stimulated for 4h with PMA and ionomycin and IRF4 expression was determined by intracellular staining and flow cytometry (MFI, mean fluorescence intensity). (**B**) Gating strategy: Cells were successively gated for leucocytes, single cells, βTCR^{+} cells with exclusion of dead, CD45.1^{+}, CD11b^{+} and CD19^{+} cells, for non-vascular (CD45i.v.$^{-}$) cells, and for CD4^{+} cells. CD4^{+} T cells were divided into *Irf4*$^{+/fl}$×*CreER*^{T2} (CD90.1^{+}) and *Irf4*$^{-/fl}$×*CreER*^{T2} (CD90.1^{-}) cells, as well as GFP^{+} and GFP^{-} cells, and all populations were analyzed for cytokine of RORγt expression. (**C**) T cells were analyzed after treatment as described in Figure 2B. % *Irf4*$^{+/fl}$×*CreER*^{T2} (CD90.1^{+}) and *Irf4*$^{-/fl}$×*CreER*^{T2} (CD90.1^{-}) of CD4^{+} T cells in the transferred population, and in spleen mLN and colon at time point of analysis. Data correspond to Figure 2C. (**D**) Representative TNF-α and IFN-γ staining of CD4^{+} T cells from mLN of infected mice after stimulation with PMA and ionomycin. ($p^{****} < 0.0001$)

Suppl. Figure 3

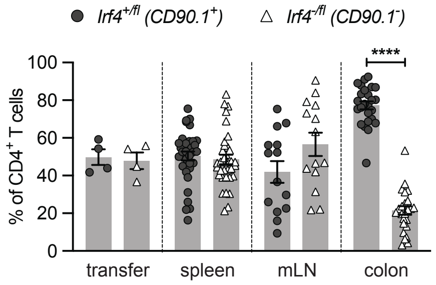

Suppl. Figure 3. Loss of IRF4 does not prevent Th17 cell maintenance.
T cells were analyzed after treatment as described in Figure 3A. % *Irf4*$^{+/fl}$×*CreER*^{T2} (CD90.1^{+}) and *Irf4*$^{-/fl}$×*CreER*^{T2} (CD90.1^{-}) of CD4^{+} T cells in the transferred population and at time point of analysis. Data correspond to Figure 3B. Mean ± SEM, results were analyzed with paired t test. (****$p < 0.0001$)

Suppl. Figure 4

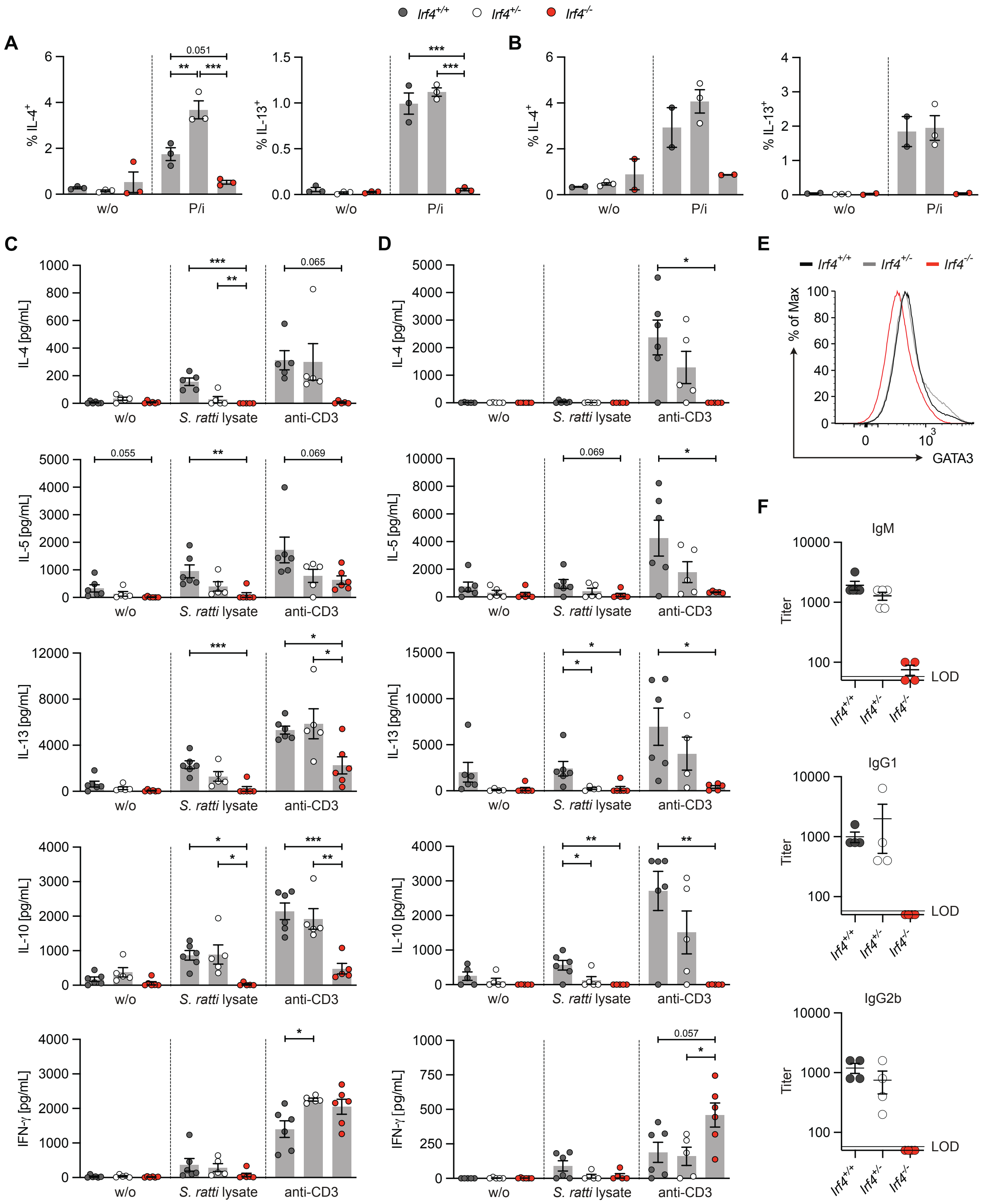

Suppl. Figure 4. IRF4-deficient mice show impaired Th2 cell response to intestinal *Strongyloides ratti* infection. *Irf4*+/+, *Irf4*+/- and *Irf4*-/- mice were infected with 2000 *S. ratti* L3 as shown in Fig. 4A. On day 10, the cytokine expression of CD4+ T cells from spleen (**A**) and mLN (**B**) was determined after incubation with PMA and ionomycin (P/i) or without stimuli (w/o) for 4h by flow cytometry. (**C, D**) On day 6, cells from spleen (**C**) and mLN (**D**) were incubated with *S. ratti* lysates, anti-CD3 mAb or without stimulation (w/o). After 3 days, cytokines in supernatants were determined with ELISA. N = 4-6 per group. One of 3 independent experiments is shown. Mean ± SEM, results were analyzed with one-way ANOVA with Tukey's multiple comparisons test. (**E**) On day 10, cells from mLN were intracellularly stained for GATA3 and analyzed with FACS. Representative histogram of GATA3 expression in CD4+ T cells. (**F**) On day 21, serum titers of *S. ratti*-specific IgM, IgG1 and IgG2b were determined. Mean ± SEM, N = 4 per group, line indicates the limit of detection (LOD). One of two independent experiments is shown. (*p < 0.05; **p < 0.01; ***p < 0.001)

Suppl. Figure 5

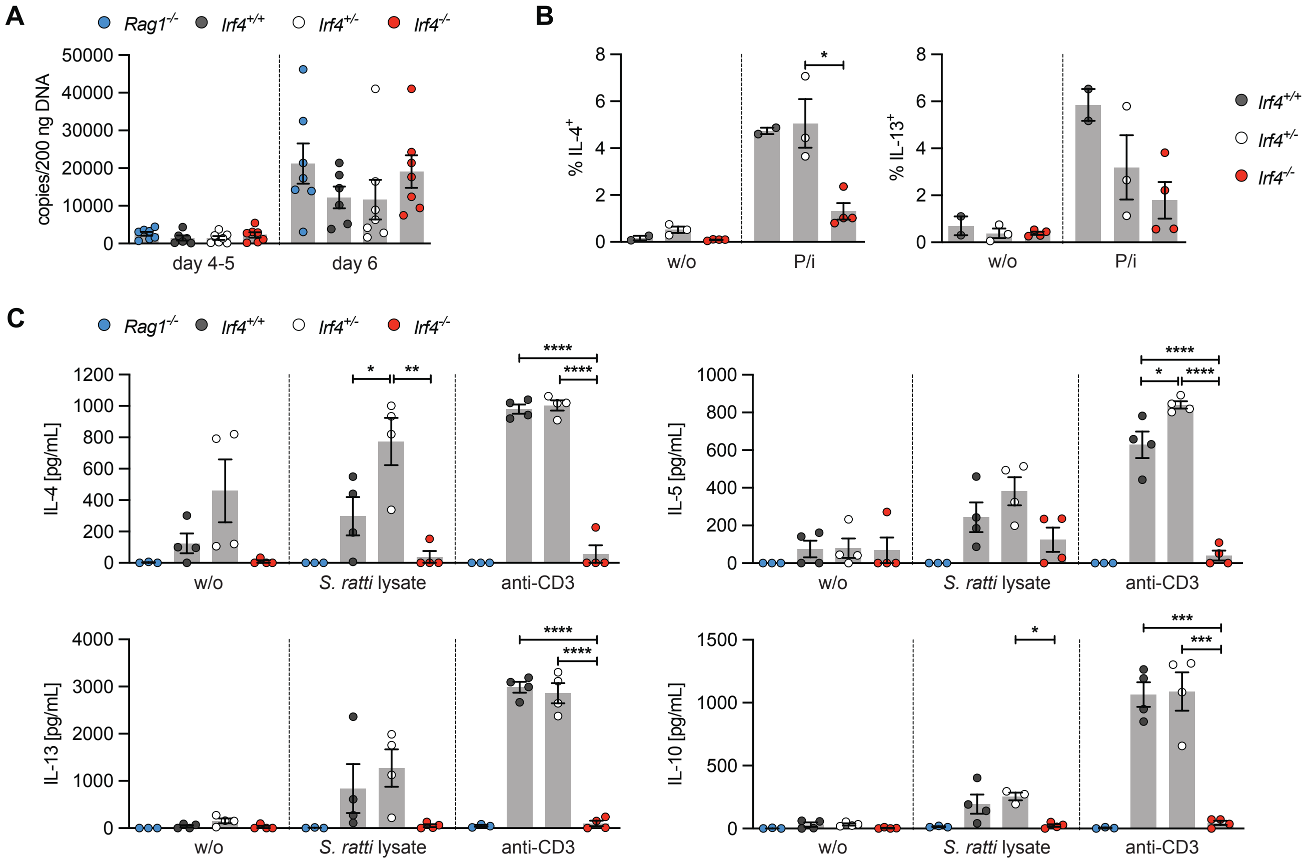

Suppl. Figure 5. IRF4-deficient T cells fail to generate a Th2 cell response to intestinal *Strongyloides ratti* infection. Naive *Rag1*$^{-/-}$ mice were reconstituted, infected, and analyzed as described in Figure 5A. (**A**) Quantitative PCR for *S. ratti*-derived DNA out of feces collected at the indicated time points. (**B**) Spleen cells from infected mice were incubated with PMA and ionomycin (P/i) or without (w/o) stimulation and the production of IL-4 and IL-13 by CD4^{+} T cells was determined by low cytometry. Data are representative of two independent experiments with each 6-7 mice/group. Mean ± SEM, results were analyzed with one-way ANOVA with Tukey's multiple comparisons test. (**C**) Spleen cells were incubated with *S. ratti* lysate, anti-CD3 mAb or without any stimulation (w/o). After 3 days, cytokines in supernatants were determined with ELISA. One of 2 independent experiments. Mean ± SEM, results were analyzed with one-way ANOVA with Tukey's multiple comparisons test. (*$p < 0.05$; **$p < 0.01$; ***$p < 0.001$; ****$p < 0.0001$)

Suppl. Figure 6

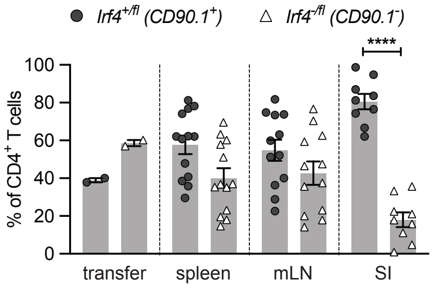

Suppl. Figure 6. IRF4 is not essential for the maintenance of Th2 cells.
T cells were analyzed after treatment as described in Figure 6A. % *Irf4*$^{+/fl}$×*CreER*^{T2} (CD90.1$^+$) and *Irf4*$^{-/fl}$×*CreER*^{T2} (CD90.1$^-$) of CD4$^+$ T cells in the transferred population and at time point of analysis. Data correspond to Figure 6B. Mean ± SEM, results were analyzed with paired t test. (****$p < 0.0001$)

Suppl. Figure 7

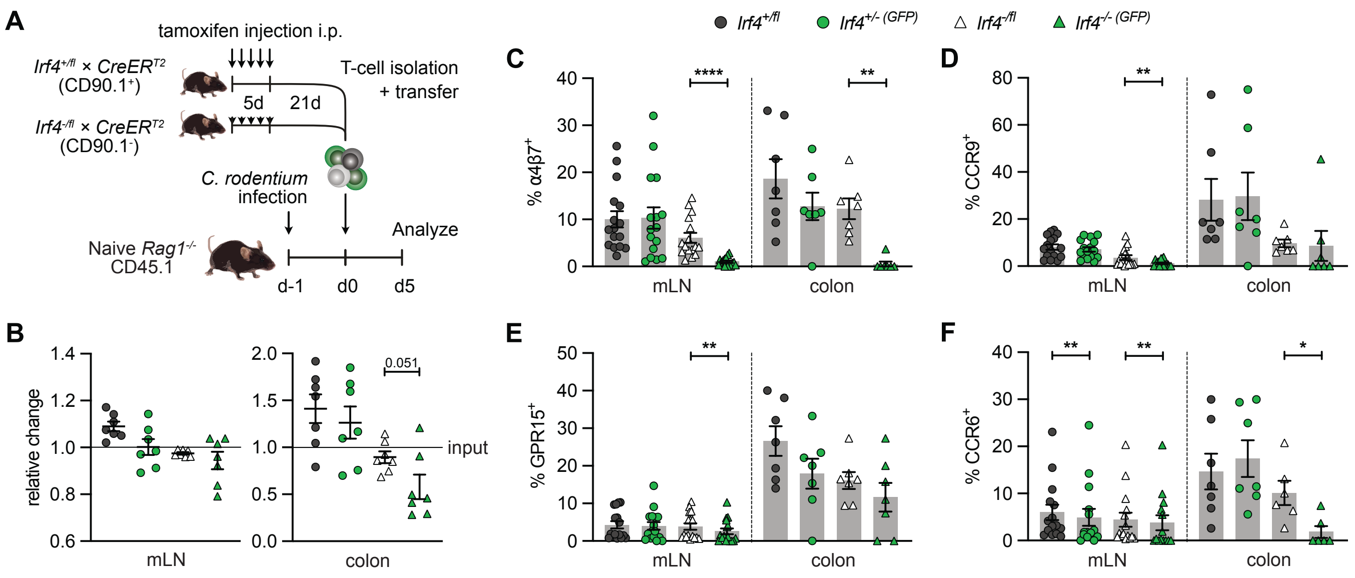

Suppl. Figure 7. IRF4-deficient CD4^{+} T cells show reduced expression of intestinal homing receptors.
(**A**) Experimental set up: naive *Irf4*$^{+/fl}$×*CreER*^{T2} (CD90.1^{+}) and *Irf4*$^{-/fl}$×*CreER*^{T2} (CD90.1^{-}) mice were treated for 5 consecutive days with tamoxifen. After 3 weeks, 4 × 10^{5} T cells from each group of mice were transferred into *Rag1*$^{-/-}$ mice, which had been infected orally with *C. rodentium* the day before. After further 5 days, mice were analyzed. (**B**) Relative change of the *Irf4*$^{+/fl}$×*CreER*^{T2}, *Irf4*$^{+/-(GFP)}$×*CreER*^{T2}, *Irf4*$^{-/fl}$×*CreER*^{T2} and *Irf4*$^{-/-(GFP)}$ ×*CreER*^{T2} CD4^{+} T cell populations determined by % cells of CD4^{+} T cells at time point of analysis divided by the % cells of transferred CD4^{+} T cells. Results of one experiment with 7 mice/group are shown. (**C** - **F**) Frequencies of α4β7^{+} (**C**), CCR9^{+} (**D**), GPR15^{+} (**E**) and CCR6^{+} (**F**) CD4^{+} T cells from mLN and colon of infected mice. Pooled results of two independent experiments with each up to mice/group. Results for colon only displaying one experiment with up to 7 mice/group.

Supplementary Table

Antibodies		Source	Identifier	RRID
anti-CCR6/CD196 (clone 140706)	V450	BD Bioscience	Cat#: 564736	AB_2738926
anti-CCR9/CD199 (clone CW-1.2)	PECy7	BioLegend	Cat#: 128712	AB_10933082
anti-CD11b (clone M1/70)	BV510/V500	BioLegend	Cat#: 101245	AB_2561390
anti-CD11b (clone M1/70)	FITC	BD Bioscience	Cat#: 553310	AB_396679
anti-CD19 (clone 6D5)	BV510/V500	BioLegend	Cat#: 115545	AB_2562136
anti-CD19 (clone eBio1D3)	PECy7	eBioscience	Cat#: 25-0193-81	AB_657663
anti-CD3 (clone 17A2)	BV421	BioLegend	Cat#: 100228	AB_2562553
anti-CD4 (clone RM4-5)	APC	BioLegend	Cat#: 100516	AB_312719
anti-CD4 (clone RM4-5)	PerCP	BioLegend	Cat#: 100538	AB_893325
anti-CD4 (clone RM4-5)	AF700	BioLegend	Cat#: 100536	AB_493701
anti-CD4 (clone RM4-5)	V450	BioLegend	Cat#: 100543	AB_10898318
anti-CD45 (clone 30F-11)	PerCP	BioLegend	Cat#: 103130	AB_893339
anti-CD45.1 (clone A20)	BV510/V500	BioLegend	Cat#: 110741	AB_2563378
anti-CD8α (clone 53.6-7)	BV650	BioLegend	Cat#: 100742	AB_2563056
anti-CD8α (clone 53.6-7)	PE	BioLegend	Cat#: 100708	AB_312747
anti-CD8α (clone 53.6-7)	PerCP	BioLegend	Cat#: 100732	AB_893423
anti-CD90.1 (clone HIS51)	PE	eBioscience	Cat#: 12-0900-81	AB_465773
anti-CD90.1 (clone HIS51)	V450	eBioscience	Cat#: 48-0900-82	AB_1272254
anti-CD90.1 (clone HIS51)	FITC	eBioscience	Cat#: 11-0900-81	AB_465151
anti-CD90.1 (clone OX-7)	APCCy7	BioLegend	Cat#: 202520	AB_2303153
anti-CD90.1 (clone OX-7)	BV786	BD Bioscience	Cat#: 740917	AB_2740559
anti-CD90.2 (clone 53-2.1)	PE	BioLegend	Cat#: 140308	AB_10641145
anti-GATA3 (clone 16E10A23)	V450	BioLegend	Cat#: 653814	AB_2563221
anti-GPR15/BOB (clone S15042I)	APC	BioLegend	Cat#: 154606	AB_2734230
anti-IFN-γ (clone XMG1.2)	APC	BioLegend	Cat#: 505814	AB_493314
anti-IFN-γ (clone XMG1.2)	APCCy7	BD Bioscience	Cat#: 561479	AB_10898181
anti-IL-13 (clone eBio13a)	V450/V488	eBioscience	Cat#: 48-7133-82	AB_11219690
anti-IL-17A (clone TC11-18H10.1)	V450	BioLegend	Cat#: 506926	AB_2632611
anti-IL-4 (clone 11B11)	APC	BioLegend	Cat#: 504106	AB_315320
anti-IRF4 (clone 3E4)	PECy7	eBioscience	Cat#: 25-9858	AB_2573558
anti-IRF4 (clone 3E4)	PE	eBioscience	Cat#: 12-9858-82	AB_10852721
anti-RORγt (clone Q31-378)	PE	BD Bioscience	Cat#: 562607	AB_11153137
anti-RORγt (clone Q31-378)	BV785	BD Bioscience	Cat#: 564723	AB_2738916
anti-TCRβ (clone H57-597)	BV605	BioLegend	Cat#: 109241	AB_2629563
anti-TNF-α (clone MP6-XT22)	APC	BioLegend	Cat#: 127614	AB_2227348
anti-TNF-α (clone MPG-XT22)	V450/BV421	BD Bioscience	Cat#: 560655/ 563387	AB_2738173
anti-TNF-α (clone MPG-XT22)	PECy7	BioLegend	Cat#: 506324	AB_2256076
anti-α4β7 (LPAM-1) (clone DATK32)	PE	BD Bioscience	Cat#: 553811	AB_395066

Suppl. Table 1. Used monoclonal antibodies for FACS-analyses.
